# Genome-Wide Analysis Reveals Diversity of Rice Intronic miRNAs in Sequence Structure, Biogenesis and Function

**DOI:** 10.1371/journal.pone.0063938

**Published:** 2013-05-22

**Authors:** Yong-ao Tong, Hua Peng, Cheng Zhan, LinHong Fan, Taobo Ai, Shenghua Wang

**Affiliations:** Key Laboratory of Bio-resources and Eco-environment, Ministry of Education, College of Life Science, Sichuan University, Chengdu, Sichuan, China; University of Georgia, United States of America

## Abstract

Intronic microRNAs (in-miRNAs) as a class of miRNA family that regulates gene expression are still poorly understood in plants. In this study, we systematically identified rice in-miRNAs by re-mining eight published small RNA-sequencing datasets of rice. Furthermore, based on the collected expression, annotation, and putative target data, we investigated the structures, potential functions, and expression features of these in-miRNAs and the expression patterns of their host genes. A total of 153 in-miRNAs, which account for over 1/4 of the total rice miRNAs, were identified. In silico expression analysis showed that most of them (∼63%) are tissue or stage-specific. However, a majority of their host genes, especially those containing clustered in-miRNAs, exhibit stable high-level expressions among 513 microarray datasets. Although in-miRNAs show diversity in function and mechanism, the DNA methylation directed by 24 nt in-miRNAs may be the main pathway that controls the expressions of target genes, host genes, and even themselves. These findings may enhance our understanding on special functions of in-miRNAs, especially in mediating DNA methylation that was concluded to affect the stability of expression and structure of host and target genes.

## Introduction

MicroRNAs (miRNAs) are ∼22 nt endogenous small RNAs that have gained the worldwide attention from researchers in gene expression regulation and gene therapy because of their vital role in the regulation of development, metabolism, stress responses, and pathogen responses of unicellular [1] and multicellular organisms [2]. Currently, a large number of miRNAs have been discovered and deposited in the public miRBase database (Release 16.0) [3]. Most of these miRNAs are located in intergenic regions between two protein coding genes and expressed as an independent transcription unit. The primary miRNA (pri-miRNA) produced by RNA polymerase II is processed into an miRNA precursor (pre-miRNA) by an RNA III enzyme, Dicer-like in plants (Drosha in animals). Then, pre-miRNA is further cut into miRNA/miRNA* duplex by Dicer-like (Dicer in animals) [4], [5]. In plants, both strands of the miRNA/miRNA* duplex are often protected from degradation through the methylation of their 3′ end [6], [7]. Finally, one strand of the duplex, which serves as the guide strand, is incorporated into an RNA-induced silencing complex (RISC), which leads to the degradation or translational repression of the target mRNAs [8], [9], [10].

However, some miRNAs, known as intronic miRNAs (in-miRNAs), are located in the intronic regions of protein-coding genes (host genes). The expression of in-miRNA is usually considered to be associated with its host gene [11], [12], [13]. Therefore, both the transcription and subsequent splicing events of the host gene affect the expression pattern of in-miRNAs [14]. However, some animal in-miRNAs have their own promoters [15], [16], [17] that make the transcription and processing of their own pri-miRNA sequences an independent transcription unit just like intergenic miRNA genes. In another class of particular in-miRNAs, mirtrons (certain debranched introns with the structural features of pre-miRNAs) are generated even without the Drosha-mediated cleavage step [18]. Therefore, the biogenesis of in-miRNAs seems to be more complex than that of intergenic ones.

Several in-miRNAs have been discovered in animal miRNAs. For instance, Rodriguez et al. (2004) identified 90 in-miRNAs of protein-coding genes, which comprises 38.8% of the 232 known mammalian miRNAs [11]. Li et al.(2007) predicted 120 in-miRNAs in humans and 76 in mice [19]. Our count of miRNAs from some species deposited in the miRBase (version 16) [3] indicates that 48% (506/1049) of intronic pre-miRNAs appear in humans, 38% (67/176) in *Drosophila melanogaster*, and 22% (39/175) *in Caenorhabditis elegans*. However, in-miRNAs in plants have rarely been systematically studied in previous research.

The fact that in-miRNAs can survive together with their host genes indicates that they may have important functions in the metabolism, growth and development, or environmental adaptation of organisms. Otherwise, in-miRNAs cannot be preserved in their long evolutionary process. However, a few studies about the peculiar functions of in-miRNAs have been reported. Barik (2008) discovered that an in-miRNA (miR-338) transcribed from an apoptosis-associated tyrosine kinase (AATK) gene effectively stimulates neural differentiation by silencing a family of genes that are functionally antagonistic to the host gene, *AATK* [20]. Rajagopalan et al. (2006) proposed that the intronic miR838 of Arabidopsis can maintain the homeostasis of its host gene, *DCL1*, through a self-regulatory mechanism [21]. Ning et al. (2011) found that miR-483-5p within the second intron of the *insulin-like growth factor* (*Igf2*) gene targets the *suppressor of cytokine signaling 3* (*Soc3*) that may be *Igf2*′s partner in fat metabolism in mice [22]. Moreover, a recent study showed that in-miRNAs can support their host genes by mediating synergistic and antagonistic regulatory effects [23]. Therefore, a detailed study on in-miRNAs, including identification of more in-miRNAs, investigation of their expression pattern, and their relationships with the host genes, is necessary to better understand in-miRNAs.

Rice is a model plant in monocotyledons and one of the most important food crops in the world. A total of 437 pre-miRNAs have been identified from cloning experiments and deep sequencing data in recent years and were registered in the miRBase (version 16.0) [24], [25], [26], [27]. In this study, we identified 74 novel miRNAs from eight published small RNA-sequencing datasets by systematical investigation of intronic regions of protein coding genes in the rice genome. These miRNAs may regulate the gene expression at both the post-transcriptional level by mRNA cleavage and the transcriptional level through a DNA methylation pathway. Expression analysis shows that most in-miRNAs (∼63%) are tissue or stage-specific, whereas the majority of the host genes exhibit stable high-level expressions, especially those harbor more pre-miRNAs. In-miRNAs and their host genes have complex and diverse regulation relationships.

## Materials and Methods

### Data Source Preparation

Rice intron sequences were downloaded from the MSU Rice Genome Annotation Project (Release 6.0; http://rice.plantbiology.msu.edu/) [28]. The sequences of precursors and mature miRNAs were obtained from the miRBase (version 16.0; http://www.mirbase.org) [3]. Eight small RNA sequencing datasets were downloaded from the NCBI Gene Expression Omnibus (GEO) repository and integrated into a local Mysql database with our custom scripts (Table S1). The datasets include GSM722128 for tricellular pollen, GSM278571 for 1–5 day after fertilization (DAF) grains, GSM278572 for 6–10 DAF grains, GSM455965 for flag leaves, GSM520640 for 3-week-old seedlings, GSM489087 for another set of 3-week-old seedlings, GSM361264 for 4-week-old seedlings, and GSM417540 for a mixture sample in calli, young buds, young roots, shoots, flowering panicle, filling panicle, and booting panicle. The number of reads for each small RNA signature was normalized into 2-fold RPM (Reads Per Million total reads) when the comparison of in-miRNA expressions among multiple samples was performed.

A total of 513 microarray datasets (Table S2) produced from 191 experimental samples were retrieved from the rice array database (http://www.ricearray.org) [29]. The data from the rice array database had been normalized so they could be used directly in our analysis. The average gene expression values were calculated from all datasets. The average expression value of probes was used for genes with multiple probes in the microarray data.

### Identification of the Inverted Repeat and miRNA/miRNA* Duplex in Introns

First, the whole intronic sequences of rice were aligned using Bl2seq [30], a utility used in comparing two sequences based on blastn or blastp approach with its reverse complementary sequence (RCS). The parameters of Bl2seq are “-p blastn -D 1 -S 1”. The hits from the Blast results were clustered into candidate fragments, and then a fragment was taken as an inverted repeat (IR) if it was more than 50 nt and the hit regions accounted for 30% of its length. The longest inverted repeat (IR) was saved if two or more IRs were actually mapped to the same locus in the genome when importing IR data into the local Mysql database. Then, the IRs were filtered by small RNA fragments from the sequencing data. The IR was considered expressed when the total number of reads of all matched small RNA fragments was 100 or more. Finally, the secondary structures of expressed IRs were further assessed using RNAFold (version 1.8) [31]. The IRs were taken as pre-miRNA candidates if they had good hairpin structures.

To extract miRNA from pre-miRNAs, the custom script scanned each sequenced RNA fragment mapped on pre-miRNAs to find out its complementary strand (with two nucleotide overhangs on the 3′ end) in hairpin structure. Subsequently, these fragments were ranked by their read numbers and were output with a threshold: the fragment or its complementary strand gives more than 20 reads in the expression level. Finally, we selected one or more fragments (some precursors can produce multiple miRNAs) as miRNAs from ranked list. Some mature miRNAs can be mapped to multiple loci in the whole genome, which may lead to a difficulty in determining their true positions. In such cases, distinct reads of miRNA* become critical.

The criteria for the miRNA annotation state that all reads are conclusively derived from randomly degraded RNA if more than 25% of reads mapped to the putative miRNA hairpin do not correspond to an miRNA/miRNA* duplex [32]. Most known miRNAs comply with this rule. However, a part of small RNAs with low expression levels does not meet this condition. We collected 80 known miRNAs with a single hit in the genome and investigated their expression data using multiple sequencing samples to obtain a more appropriate percentage. Thirty-eight of these miRNAs with a low-level mature miRNA expression value were then removed. The read ratio of the miRNA/miRNA* duplex in the hairpin of the remaining miRNAs are listed in Table S3. In Table S3, the ratio ranges from 44.44% to 100%. We set the threshold of the read ratio to 40% instead of the original 75% to adopt a relatively loose strategy for miRNA annotation. The read ratio of our novel miRNAs are listed in Table S4.

To exclude siRNAs from our results, we first removed candidate precursors with continuous reads which indicates a low cleavage precision, and then removed the candidates with abundant sequencing reads (more than 10% total unique reads) in their anti-sense strands. Furthermore, the bulges located in miRNA/miRNA* duplex eliminated the possibility of siRNAs from hairpin RNAs (Figure S2).

### Conservation Test and Chip-seq Dataset Preparation

The conservation analysis of novel miRNAs was conducted through the interface of remoteBlast in Bioperl (E-value: 5E-3) in the following seven plant species: *Arabidopsis thaliana*, *Hordeum vulgare*, *Panicum virgatum*, *Saccharum officinarum*, *Sorghum bicolor*, *Triticum aestivum*, and *Zea mays* [33].

We downloaded peak data from two Chip-seq datasets GSM756374 and GSM756375 (11 days rice seedlings) and mapped signal peaks to host gene loci with Perl scripts.

### Target Prediction and Gene Ontology (GO) Enrichment Analysis

Potential mRNA targets for miRNAs or siRNAs were predicted using an online program (http://mpss.udel.edu/rice_pare/index.php?menu=https://raichu.dbi.udel.edu/~apps/tp/index.php?SITE=rice_pare) based on an algorithm described by Jones-Rhoades and Bartel [34]. The following default parameters were used in our analysis: (1) The score is less than or equal to 2.5 (a mismatch is given a score of 1, a wobble (G:U mismatch) is given a score of 0.5, and a bulge is given a score of 2); (2) A perfect match is required at 10 or 11 nt positions. (3) No more than one mismatch is allowed at the 2 nt to 9 nt positions. The results which mapped to intergenic regions or a noncoding gene locus were not considered in this study.

The GO analysis of putative targets was conducted using the ClueGO plug-in in Cytoscape [35], [36]. The GO annotation sources were extracted and created from our local rice gene annotation database described in [37]. In the ClueGo settings, the statistical test used Enrichment option with a Benjamini-Hochberg PV correction. The GO term restriction settings were set to level 3–8 and at least 2 genes or 4% genes in each GO term. The Kappa score was set to 0.3. GO term grouping was used with an initial group size of 3 and 50% for the group merge. The GO terms with a corrected P-value<0.05 from the ClueGO results were saved.

### Expression Analysis of Host Genes

First, we removed genes with no introns from a total of 47652 gene models according to the gene annotation from the MSU Rice Genome Annotation Project (Release 6.0; http://rice.plantbiology.msu.edu/) [28] to eliminate the effect on gene expression from the introns themselves. The expression data of the remaining 43031 gene models (intron-containing genes) were retrieved from the microarray data described above.

We randomly extracted 1000 genes from the intron-containing genes with 1000 iterations and plotted their expression distributions from 0 to 16 with 8 intervals using the R software to test the expression distribution of the randomly sampled genes.

Microarray datasets from the following five samples were used to study the relationship between in-miRNAs and their host genes: mature leaves (GSM357682, GSM357683, GSM357684); 0–2 DAP (GSM159207, GSM159208); 3–4 DAP (GSM159211, GSM159212); 5–10 DAP (GSM159213, GSM159214, GSM159215); and 4-week-old seedling shoots (GSM470666, GSM470667, GSM470765, GSM470766). Each sample had two or three repeats and the average expression values were calculated for further analysis. Data from 0–2 and 3–4 DAP were combined as a new 0–4 DAP dataset with an average expression value. The Spearman correlation coefficients between miRNAs and their host genes were calculated using the R software.

### K-means Clustering (KMC) of in-miRNAs

The number of reads for all in-miRNAs were first normalized into 2-fold RPM and then transformed into log2. The expression data were analyzed in MeV, an open-source genomic analysis program created by the Mev Development Team (http://www.tm4.org). The distance metric for KMC [38] is the Euclidean Distance and the number of clusters is set to 10. The parameter of maximum iterations is set to 50.

### Detections of Novel pre-miRNAs and Mature miRNAs

For pre-miRNAs' detections, total RNAs were extracted from 3-week-old rice seedlings and tricellular pollen with RNAprep pure Plant Kit (TIANGEN, BeiJing, CN) and transformed into cDNA with PrimeScript®RT reagent Kit (Takara). The PCR reactions were performed in the following cycle conditions: 94°C, 5min; 94°C, 30 sec; 48°C, 20sec; 72°C, 20sec for 38 cycles; 72°C, 10 min. All primers used to detect pre-miRNAs were listed in Table S5. The electrophoresis uses 2% agarose gel.

For detections of mature miRNAs, total RNAs were extracted from 3-week-old rice seedlings with Trizol (Invitrogen). The experimental workflows generally follow the methods described in Wang et al [39], however, we use biotin instead of fluorescein. The designed probes were shown in Table S6.

## Results

### Novel miRNAs in Intronic Regions of the Rice Genome

**Figure 1:**
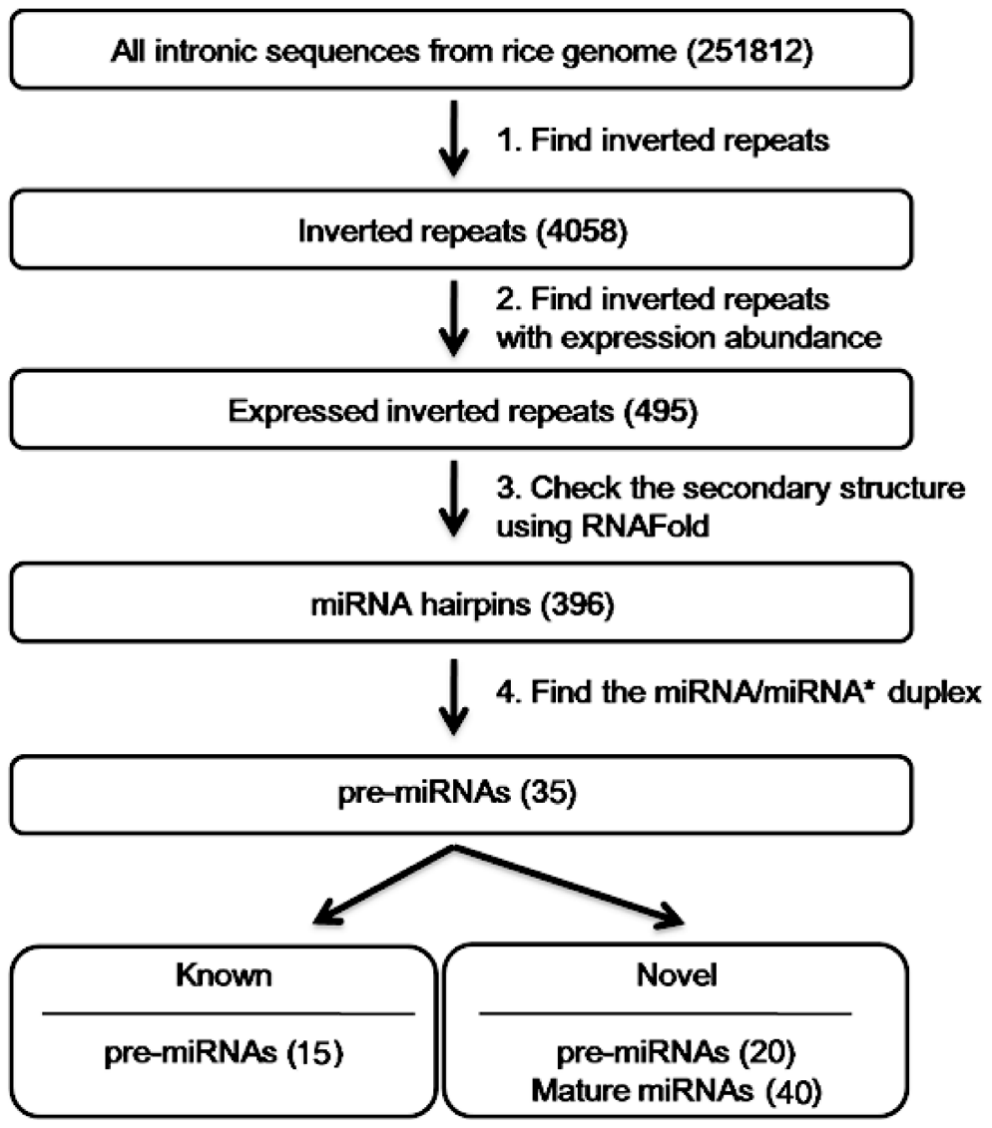
The workflow for identification of intronic miRNAs in rice genome. In each step, the numbers of the sequences are indicated in parenthesis. At the last step, 'Known' indicates known pre-miRNAs found in our study, while 'Novel' indicates novel pre-miRNAs discovered by our methods.

The strategy used in this paper in identifying miRNAs in the intronic regions of the rice genome is as follows: (a) The IRs were picked up from the intronic sequences of the rice genome downloaded from the MSU Rice Genome Annotation Project database. (b) The IRs with low-abundant expressions (less than 100 reads) were filtered out by checking their total reads from all eight sequencing samples. (c) The secondary structures of IRs were assessed using RNAFold (version 1.8) [31]. (d) Mature miRNA and miRNA* (absent in a few case) were extracted from pre-miRNA candidates using our custom script. The whole workflow for the identification of miRNAs is shown in Figure 1.

A total of 396 candidate hairpins with expressed small RNA fragments (18 nt to 25 nt) were obtained from the intron data of the rice genome after three steps of filtering (Figure 1). However, only 35 hairpins were considered pre-miRNAs (Table S7), which fit the following criteria for annotation of plant miRNAs: (a) miRNA and miRNA* form the duplex with two nucleotide overhangs on the 3′ end; (b) no more than four mismatches in the miRNA/miRNA* duplex; and (c) asymmetric bulges are minimal in size (one or two bases) and frequency (typically one or less) within the miRNA/miRNA* duplex [32]. These pre-miRNAs include 20 novel pre-miRNAs and 15 known pre-miRNAs that produce 40 and 41 mature miRNAs, respectively.

**Figure 2:**
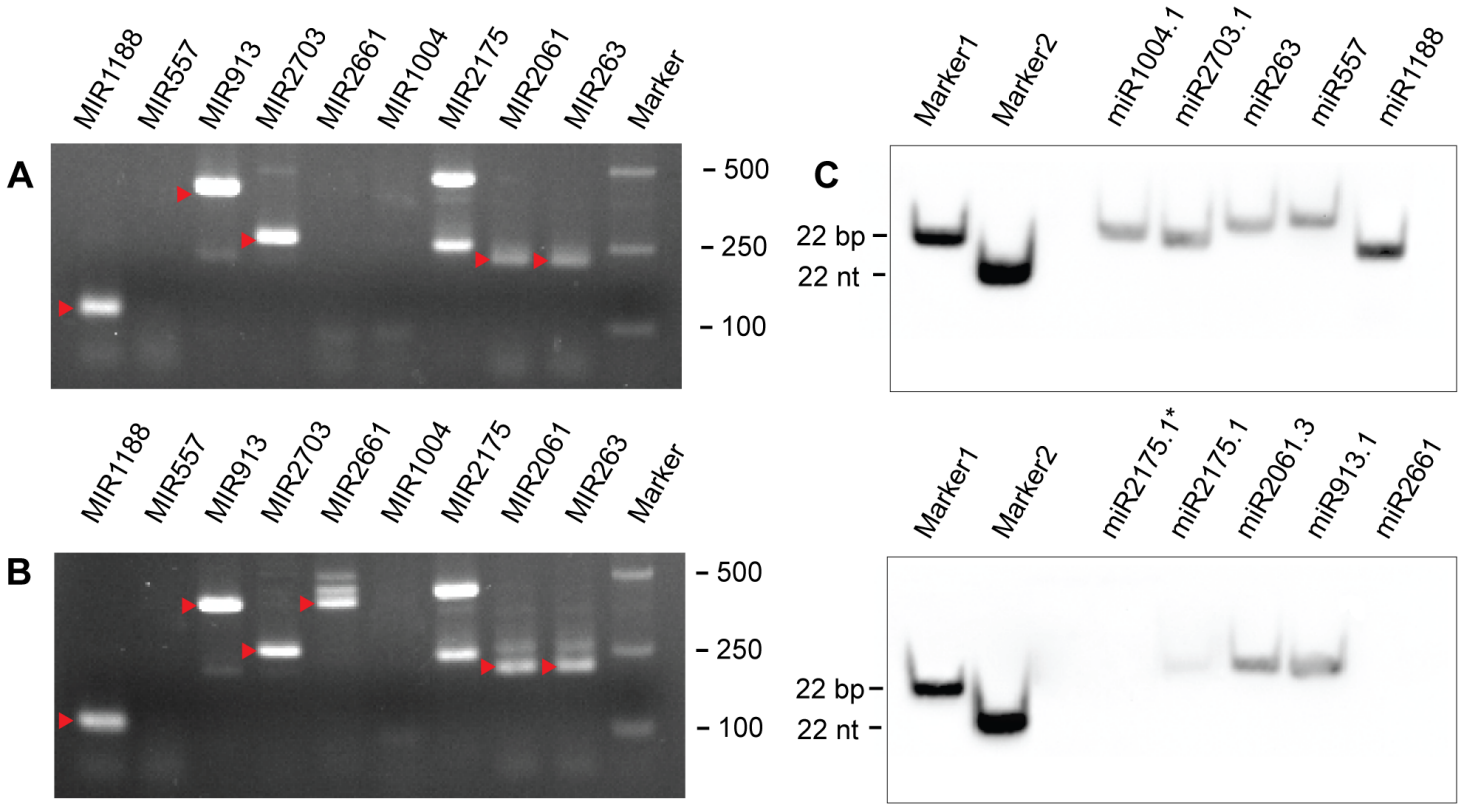
Detections of novel pre-miRNAs and mature miRNAs. Reverse transcriptase-PCR of nine novel pre-miRNAs in 3-week-old rice seedlings (A) and tricellular pollen (B). The target bands are indicated by red arrows. (C) Detections of ten mature miRNAs using Liquid northern hybridization. A probe-DNA hybrid duplex (Marker1, 22bp) and a single probe (Marker2, 22nt) are used as markers.

To test the accuracy of our predictions, we selected nine novel pre-miRNAs to preform experimental validations. MIR913 and MIR2661 were chose because of the long length and obvious expression in pollen; other seven pre-miRNAs were randomly selected from pre-miRNAs with expression in 3-week-old rice seedlings. As shown in Figures 2A and 2B, six pre-miRNAs (66.7%) have been detected in RT-PCR. A pollen specific MIR2661 only have a target band in tricellular pollen samples (Figure 2B). We also validated expressions of mature miRNAs from these selected pre-miRNAs in 3-week-old seedlings. Except for miR2175.1* and pollen specific miR2661, other miRNAs were detected. Especially, miR1188 show a high-level expression, as expected (Figure 2C).

**Figure 3:**
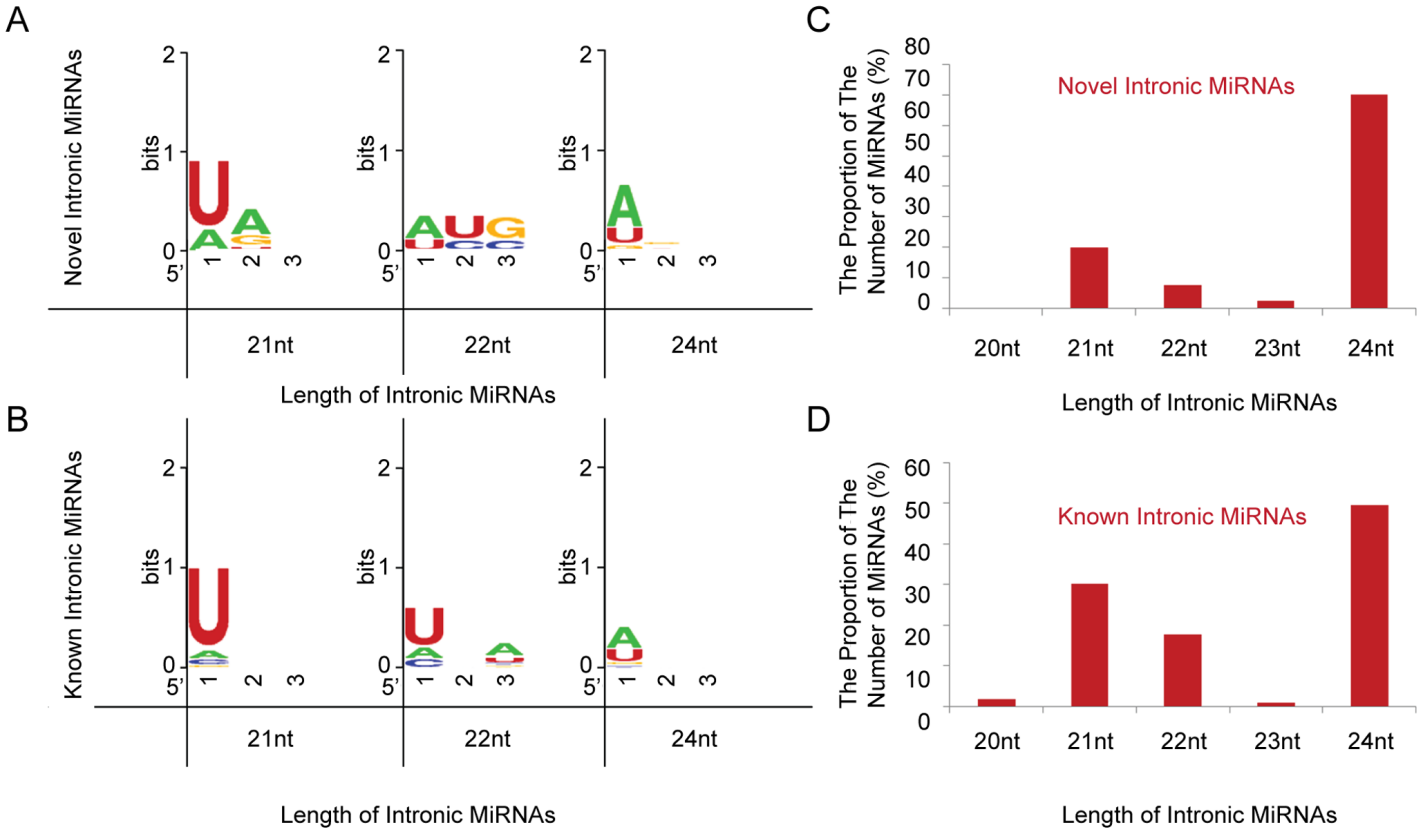
The preference for the 5′ initial base and length distribution of mature in-miRNAs. (A) and (B) Weblogos of the first three bases at the 5′ terminal of novel and known in-miRNAs. U, A, G, and C are represented in red, green, orange, and blue, respectively. (C) and (D) Length distribution of novel and known in-miRNAs.

Among the 40 newly found miRNAs, 11 (∼28%) are 20 nt to 22 nt in length, the majority of which (∼64%) initiate with a 5′ U - a preference base for Ago1 [40] and share the characteristics of the conserved miRNAs in Arabidopsis [21]; 28 miRNAs (70%) are 24 nt long but only about 25% have a 5′ U, whereas 64% have a 5′ A and 11% have a 5′ G (Figures 3A and C). On the other hand, the survey of structural characteristics for 113 in-miRNAs from the known precursors (see next section) showed that 56 miRNAs (∼50%) are 20 nt and 22 nt in length and 75% of these miRNAs initiate with a 5′ U and 14% with a 5′ A, whereas the frequency of 5′ U is only 32% and that of A is up to 54% in 56 24 nt long miRNAs (∼50%) (Figures 3B and D).

### Mining miRNAs from Known Intronic pre-miRNAs in Rice

Combined with the gene annotation data from the MSU Rice Genome Annotation Project (Release 6.0), we obtained 69 intronic pre-miRNAs that contain 79 mature miRNAs (Table S8) from the sequences for rice pre-miRNAs and mature miRNAs. Their genomic coordinates were extracted from the miRBase (Release 16.0).

**Figure 4:**
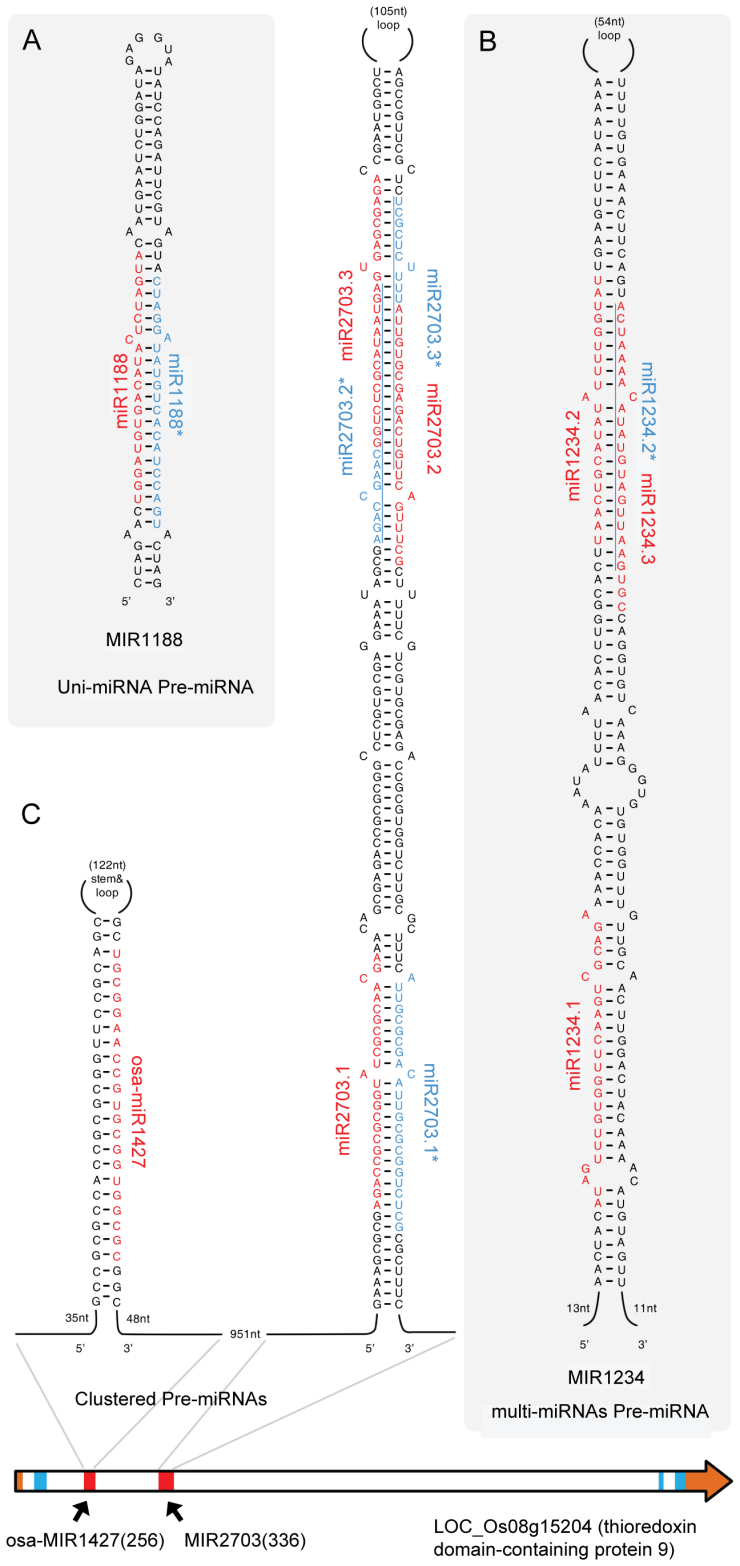
Three categories of intronic pre-miRNAs. Mature miRNAs and their miRNA*s are marked with red and blue, respectively. For some miRNA*s that overlapped with miRNAs, we use blue vertical lines to indicate their spans. (A) Uni-miRNA pre-miRNA: pre-miRNA encoding only one mature miRNA. (B) Multi-miRNAs pre-miRNA: pre-miRNA encoding more than one mature miRNA. (C) Clustered pre-miRNAs: multiple pre-miRNAs located in close genomic position to each other (<3000 nt gap). In the schematic diagram of the LOC_Os08g15204 gene structure, red, blue, and brown bars indicate pre-miRNAs, exons, and untranslated regions (UTR), respectively.

In the data processing of pre-miRNA sequences, some pre-miRNAs with tissue-specific pattern can produce two or more distinct mature miRNAs (Figure 4B; Table 2). Similar results have been reported in previous studies [5], [25], [41], [42], [43]. Some miRNAs may exist in known pre-miRNAs. Thus, we re-investigated the 69 known intronic pre-miRNAs using our custom scripts and 8 public small RNA sequencing data. As a result, another 34 mature miRNAs with an obvious expression were uncovered, resulting in 113 mature in-miRNAs derived from known pre-miRNAs (Table S8).

### The Diversity of Novel Intronic pre-miRNAs

According to the structural features of the novel intronic pre-miRNAs, we divided them into the following three categories: non-clustered uni-miRNA pre-miRNA (UMP-miRNA, one pre-miRNA can be processed into only one mature miRNA); non-clustered multi-miRNAs pre-miRNA (MMP-miRNA, one pre-miRNA can generate two or more miRNAs); and clustered pre-miRNAs (CP-miRNA, neighboring pre-miRNAs with a <3000 nt gap) (Figure 4). The detailed secondary structures of these novel pre-miRNAs are given in Figure S2.

Among the 40 novel miRNAs, 11 are derived from UMP-miRNAs (Table 1; Figures 4A and S2). The lengths of these pre-miRNAs range from 76 nt to 917 nt. Moreover, miRNA*s were detected for all miRNAs, except miR966 and miR2661, which are specifically produced from the tri-nuclear pollen sample [37] (Table 1). However, for miR2661, another adjacent RNA/RNA* pair with low reads was detected in its hairpin. Thus, we did not treat it as a miRNA*-deficient case.

**Table 1:** Eleven novel intronic miRNA precursors encoding single miRNA.

Pre-miRNA		Mature miRNA			
ID	Length	Sequence	Intron^c^	Reads	Hits
MIR53	125	UUAUAUUUUGGGAUGAAGUGA	3	93	1
MIR263^a^	405	GUGGGUCUAAGGACUAUAUUAACC	2	461	4
MIR557	84	AUUUGUUGUAUUAGGGAAUGUCUC	1	232	1
MIR851	334	AACGGCAAUGGCAUUUCUCGG	4	113	1
MIR966^b^	153	AUGACUUACAAUCUGAAACGGA	1	224	1
MIR1188	93	UGGAUGUGACAUACUCUAGUA	3	7753	1
MIR1414	76	AGCUUCUGACAGCUGCAGUUUCUC	3	1344	1
MIR2745	484	UUUGGACCGGUAUGAUACAAUAAA	4	38	1
MIR2749	119	AACCUAGUACUGAAUGUGACGCAU	1	75	1
MIR2661	917	AGGCUGUAGGAUUUGGGUUGAACC	2	283	1
MIR1181^a^	617	AAACUUUCCUGGUCGCGUCGG	3	157	2

a MiRNA sequence can be mapped to multiple sites in genome.

b MiRNA*-deficient (miRNA* strand is not detected in sequencing data).

c Which intron the miRNA locates in.

On the other hand, 29 newly found mature miRNAs (∼73%) were produced from nine MMP-miRNAs (Table 2; Figure 4B). Further analysis of the sequence structure showed that some mature miRNAs overlap, whereas others do not in their precursor sequences (Figure S2). There are also 19 MMP-miRNAs in the known intronic pre-miRNAs (Table S8). Among total 28 MMP-miRNAs, seven (e.g., osa-MIR1871, MIR2944) are processed into only one 24 nt miRNA species, three (e.g., osa-MIR1857) into only 21 nt species, ten (e.g., osa-MIR2429, osa-MIR1847) into both 21 and 24 nt species, and the remaining ones generate other species combinations, such as 20 nt/22 nt, 21 nt/22 nt, and 21 nt/22 nt/24 nt (Table 2, Table S8), indicating that pre-miRNA in introns can be processed by different classes of DCLs just like the intergenic MIR gene [41].

For the known in-miRNAs from rice, the length of their hairpin precursors is up to 361 nt (miRBase, version 16.0; http://www.mirbase.org). However, in our study, seven miRNAs, including MIR263, MIR2745, and MIR913, exceed this length (Tables 1 and 2). The longest was a pollen-specific pre-miRNA, MIR913, with a length of 1265 nt and can produce three precise mature miRNAs (83.4% total reads).

**Table 2:** Nine novel intronic pre-miRNAs encoding multiple miRNAs.

Pre-miRNA		Mature miRNA				
ID	Length	N^a^	Sequence	Intron^b^	Reads	Hit
MIR2944	508	.1	AGUGACGAGUAUGAAGAGUUGAAG	10	212	1
		.2	AUGGUAAGGAAAGGUAGCAGUAGC		141	
		.3	AAGAGUUGAAGAAGAAUGUGAUGG		182	
MIR2173	269	.1	UAGAGACAAUAAGAGAUCGGG	2	81	1
		.2	UAUGAUAGAGACAAUAAGAGA		53	
		.3	UGAUAGAGACAAUAAGAGAUCGGG		44	
MIR1004	90	.1	AGGGUAUUUUGGUAUUUUCCUGUC	1	301	1
		.2	AAUAUAUUUUGUACAAGAGAUGGG		442	1
MIR2687	229	.1	AGAAACGGCACUUGUGAAAUCCAA	9	118	1
		.2	UGAGAAACGGCACUUGUGAAAUCC		72	1
MIR913	1265	.1	AUGGUACUGUAACCAGAAGCGG	1	3458	1
		.2	UGGUACUGUAACCAGAAGCGGGC		5170	1
		.3	UCCGGCUACAGUACCAUACUAC		183	1
MIR1234	267	.1	AUAGUUUGUGGUUCAAGUCGCAGA	1	159	1
		.2	UAACUGCAUAUAUUUUGGUAU		113	1
		.3	ACUAAAACAUAUGUAGUUAAGUGC		81	1
		.4	UGUAGUUAAGUGCCAGGUGUC		47	1
MIR2703	336	.1	AGACCGCGCGGUAUCGCGCAACGA	2	621	1
		.2	AUUGUGCGAGACUGUUCAGUUUCG		671	1
		.3	UUUAUUGUGCGAGACUGUUCAGUU		461	
		.4	GUCUCGCAUAAUGAGUGAGCGAGA		187	1
MIR2061	443	.1	GAGGACAGGUGGGAUGGGAUGAUC	8	363	1
		.2	AAACCAAACACCUUGAAAAAUGGG		101	1
		.3	UGUUUGGUUUGAGGACAGGUGGGA		148	1
		.4	AGGGUGUUUGGUUUGAGGACAGGU		83	1
MIR2175	114	.1	AAAGUCAAAACGGCUUAUAAUUUG	2	198	1
		.1*	UAUUAUAAGACGUUUUGACUUUUU		327	4
		.2	UCAAAACGGCUUAUAAUUUGAAAC		163	1
		.3	AAACGGCUUAUAAUUUGAAACAGA		225	1

a N indicates the numerical suffixes of the nomenclature of mature miRNAs from the same precursor (e.g., miR2944.1 and miR2944.2).

b Which intron the miRNA locates in.

Additionally, 16 (∼17%) pre-miRNAs in the introns of the rice genome gather into seven clusters just like some intergenic miRNAs [44] (Table 3; Figure 4C). Among them, three clusters are novel. The length of cluster-containing introns ranges from 1207 to 8374. It indicates that the formation of in-miRNA clusters is not associated with intron length. In contrast to the miRNA clusters in intergenic regions which are usually composed of miRNAs from the same family [45], most of the miRNA clusters in introns come from different families, suggesting that the formation pathway of in-miRNA clusters is different with intergenic miRNA clusters.

**Table 3:** Seven pre-miRNA clusters in rice intronic regions.

Pre-miRNA Cluster^a^	Intron Length	Host Gene	Intron^c^
osa-MIR1862d^b^- osa-MIR1876 -osa-MIR1884b	2147	LOC_Os10g08930	2
osa-MIR1423^b^ **-** osa-MIR1868^b^	2470	LOC_Os04g32710	3
osa-MIR1862e - osa-MIR2872	5115	LOC_Os11g05562	3
osa-MIR1863b - osa-MIR1863c	6465	LOC_Os12g09290	1
new-MIR2173^b^-osa-MIR1870^b^-new-MIR2175^b^	2918	LOC_Os06g36160	2
osa-MIR1427^b^ - new-MIR2703^b^	8374	LOC_Os08g15204	2
osa-MIR1884a - new-MIR557^b^	1207	LOC_Os02g18660	1

a The pre-miRNA name with a prefix “new-” indicates a novel pre-miRNA.

b Novel miRNAs and known miRNAs uncovered in this paper.

c Which intron the miRNA locates in.

### MicroRNA-like sRNAs from Long Hairpins in Introns

In addition, two microRNA-like sRNAs, namely, MIR3863 and MIR2284, were identified. Like the two microRNA-like hairpins (one is the hairpin of the pre-miR436 and the other is generated from the antisense strand of LOC_Os06g21900) found by Zhu et al. [46], 11414 unique reads were generated from the 5′ and the 3′ arm of MIR3863 and only 159 reads were mapped to its antisense strand. Furthermore, 24735 unique reads were produced from the sense strand in the hairpin of MIR2284 and only 206 reads were produced from the antisense strand. However, unlike their two hairpins that only produce phased 21 nt small RNAs, our MIR3863 tends to generate 24 nt sRNAs with a 5′ A or 5′ G, and the MIR2284 seems to produce a mixture of 21 and 22 nt sRNAs with 5′ U (Table 4), suggesting that microRNA-like hairpins from introns may also be processed by multiple members of the DCL family.

**Table 4:** Two microRNA-like small RNAs from rice introns.

Pre-miRNA		Mature miRNA				
ID	Length	N^a^	Sequence	Intron^b^	Reads	Hit
MIR3863	1147	.1	AUGCCGCUGUGUACAACUGUAGUA	3	590	1
		.2	AGGCAAUUCAGAAAUUCGACUAGG		405	1
		.3	ACAGGCAAUUCAGAAAUUCGACUA		525	2
		.4	UGCUUGUGAACUACUACAUGAGUA		255	1
		.5	GUAUGUGCCUAUAAUCGCCAUGGU		250	1
		.6	AACUGUAGUAGACAAUUGCCUAUG		203	1
		.7	GAAAUUCGACUAGGUUACUGCAAA		202	1
		.8	GCUUUGCCUCGUUGGCUCACGGAU		130	1
		.9	GUAAUCAAAUUAGAGAUCAGAUUG		103	1
		.10	AGUAACCUAGUCGAAUUUAUGAAU		97	1
		.11	AGGAAGUCAAGUAAAGUAUGACAA		75	1
MIR2284	1681	.1	UAUGACUGACAUUGAUGACGAG	5	1606	1
		.2	UCACGGUUAGAUCGACGCGGG		623	1
		.3	UGACUGACAUUGAUGACGAGU		739	1
		.4	AGACGUCUGAGGAAUGUCGGCA		268	1
		.5	UGACAUUGAUGACGAGUAGAAG		254	1
		.6	UGAGAUUGACAGACGGUUUAGC		177	1
		.7	UGGAGGGUUGUCAUCGGUAUC		264	1
		.8	UGUUGCAUCGGUUUGUCGUUA		223	1
		.9	UCGCAAACUGUUGAUGCGCCC		175	1
		.10	UACACAGCAUCGUACGACGACA		141	1
		.11	UGGGUGGUCCUCGCUCGUCUGG		114	1
		.12	AGGAAACGACAAUGACCCGUGU		86	1

a N indicates the numerical suffixes of the nomenclature of mature miRNAs from the same precursor (e.g., miR2944.1 and miR2944.2).

b Which intron the miRNA locates in.

### Expressions of Most in-miRNAs are Tissue or Stage-specific

**Figure 5:**
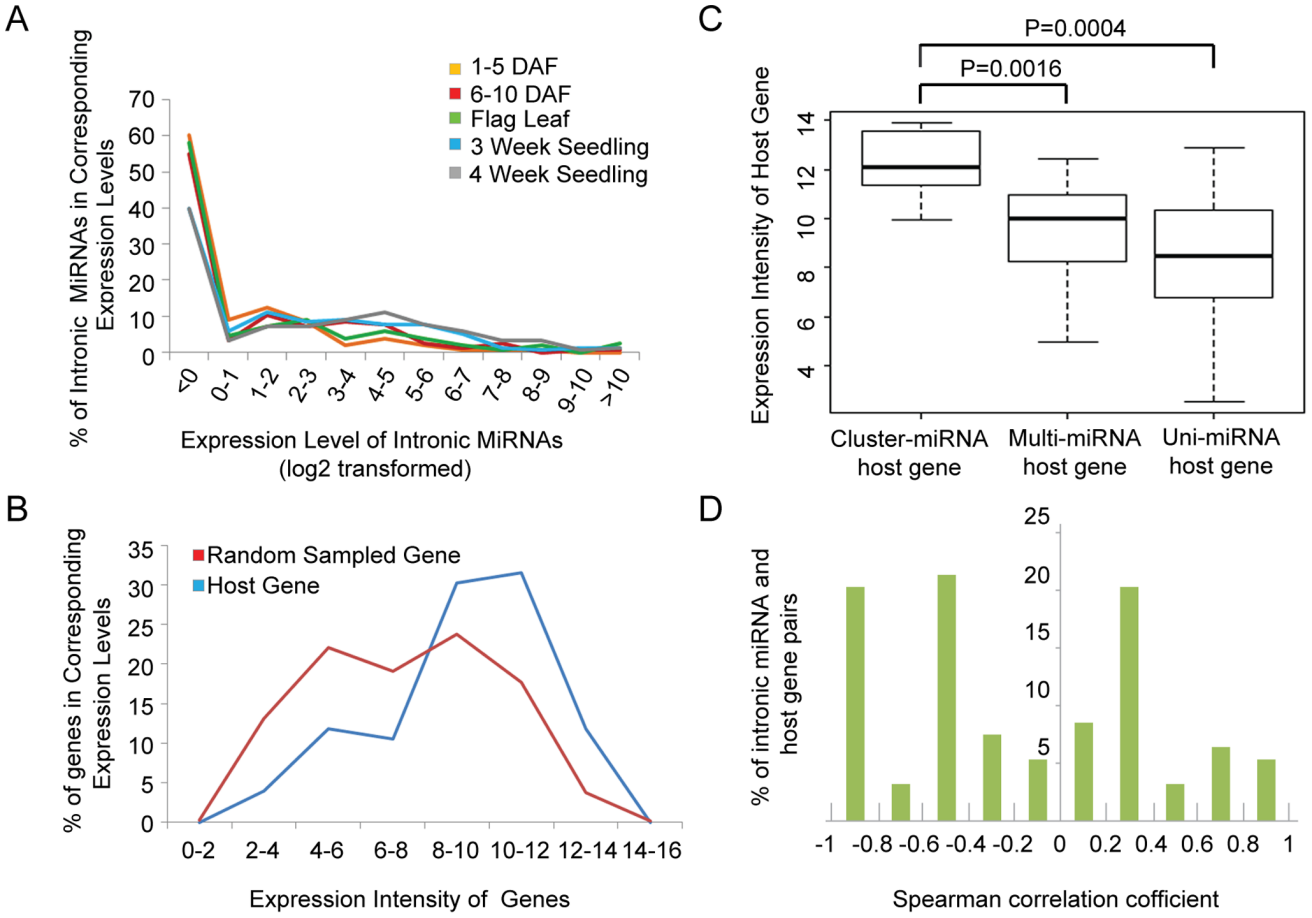
Expression of in-miRNAs and their host genes. (A) Expression of rice in-miRNAs in different tissues. (B) Expression distribution of 76 host genes and 1000 randomly sampled genes for rice in-miRNAs. The interval of expression values in the x-axis is right-closed. (C) Box plots of the expression level of three categories of host gene. The expression intensity of host genes containing miRNA clusters shows a significantly higher level than the other two categories. The P-values are calculated from a two-sided Wilcoxon rank sum test. (D) The Spearman correlation coefficient between in-miRNAs and their host genes.

We next conducted an in silico analysis of spatiotemporal expression analysis of 153 in-miRNAs (113 from the known intronic pre-miRNAs and 40 novel predicted ones). Apart from the super expression (over 500 RPM) of miRNA 167g variants and miR1188 in leaves and seedlings, most in-miRNAs have low and moderate levels of expression or no expression and generally show a declining tendency in the number of miRNA with increasing expression levels (Figure 5A, Table S9).

All in-miRNAs are partitioned into 10 groups based on their expressions using the k-means clustering algorithm (Figure S1). Forty-nine (∼32%) in-miRNAs give nearly no expression in all detection samples (Cluster 5), eight miRNAs in Cluster 1 show a stable high-level expression, and the remaining ones (∼63%) are of strong or weak spatial-temporal specificity. For instance, miRNAs in Cluster 2 show strong specific expression in 3–4-week-old seedlings, whereas miRNAs in Cluster 9 produce no expression in these stages instead of an obvious expression in the other three stages. Moreover, miRNAs from different pre-miRNAs in the same intron give different expression levels. For example, osa-miR2872 has no expression and osa-miR1862e has a moderate expression (∼42 RPM) in leaves. Furthermore, some in-miRNAs from the same precursor also exhibit a significantly different expression pattern. For instance, osa-miR1868 is expressed in grains and flag leaves but not in seedlings, whereas osa-miR1868.2 shows a high expression level in seedlings (Table S9), implying that different maturation mechanisms of intronic pri-miRNAs occur in different tissues or developmental stages.

### A Biased Expression Distribution of Host Genes

The 153 miRNAs investigated in this paper are from the introns of 80 protein-coding genes with a broad spectrum of biological roles involved in housekeeping genes, development, biotic, and abiotic stress response-related genes, such as transcription factor, F-box proteins, exosome complex exonuclease, disease resistance protein, cytochrome P450, and adaptin protein. However, four host genes, namely, LOC_Os12g28520 (abscisic acid-inducible, putative), LOC_Os03g06640 (retrotransposon protein, putative, unclassified), and two hypothetical proteins (LOC_Os07g25660, LOC_Os02g14020), have no available probes in the current affymetrix microarray data (Table S10).

Expression analysis showed that these host genes present a biased expression distribution (a peak between value 10–12) (Figure 5B). On the other hand, 1000 randomly sampled intron-containing genes (1,000 iterations) have a bimodal curve of expression (a peak at value 5 and another at value 8). The ratio of genes with high expressions was less than that of the host genes, whereas that of low and moderate expressions was higher than that of the host genes (Figure 5B).

Interestingly, further analysis indicated that the expression levels of the host genes containing CP-miRNAs are significantly higher than both the multi-miRNA genes with MMP-miRNA and the uni-miRNA genes that contain UMP -miRNA (Figure 5C).

### Expression Relationship between in-miRNAs and Host Genes

We analyzed the expression data of in-miRNAs in four similar stages to examine whether the expression of in-miRNAs is parallel to that of the host genes. The four stages are as follows: 1–5 DAF (0–4 DAP for sRNA); 6–10 DAF (5–10 DAP for sRNA); 4-week-old seedling shoots; and flag leaves. In-miRNAs not expressed in three or more samples were removed. The saved 95 pairs of miRNA-host-gene expression data were used to conduct a correlation analysis and 15% of the pairs yielded a positive correlation (correlation coefficient>0.5) and 23% yielded a negative correlation (correlation coefficient<−0.5) (Figure 5D; Table S11). That is, most of in-miRNAs have a distinct expression pattern from their host genes, indicating that the expression of in-miRNA is controlled by other mechanisms in addition to the host gene promoter.

### Independent Transcription of in-miRNAs in Rice

The expressions of some in-miRNAs are abundant while their host transcripts are not. It suggested that these in-miRNAs may have their own independent promoters. We downloaded two Chip-seq (H3K4me3) Datasets (named as HY and CHD) and obtained signal peaks located in all host genes. Most of peaks were observed in less than 1000bp downstream of transcription start site (TSS) of host genes, however, several peaks outlay (Figures S3A and B). For instance, the peak located more than 1000bp downstream from TSS of a homeobox domain containing protein (LOC_Os03g52239), might represent an independent TSS (Figure S3C). Consistent with this, LOC_Os03g52239 and its intronic miR1188 exhibited different expression patterns (Table S11). Besides, LOC_Os08g41460 also had an alternative TSS (Figure S3D).

### Function of in-miRNA Targets

About 6% of the in-miRNA targets are transcription factors, whereas others are involved in diverse functions. In-miRNAs may be non-conserved and have not evolved into having an exclusive function like conserved miRNAs during a relatively short evolution time [47]. In this study, a conservation analysis of all in-miRNAs was conducted across seven other species, namely, *A. thaliana*, *H. vulgare*, *P. virgatum*, *Saccharum*, *S. bicolor*, *T. aestivum*, and *Z. mays*. Only 26 in-miRNAs (∼17%) were found to have homologous fragments in searched species (Figure S4). Furthermore, GO analysis revealed that in-miRNA targets are enriched in several GO terms in the biological process category, such as response to extracellular stimulus, cell communication, cell death, lipid metabolic process, and protein folding (Figure S5).

Trans- or cis- DNA methylation directed by 24 nt miRNA (lmiRNA) is another important regulation pattern of gene expression discovered recently by Wu et al. (2010) [42]. Thus, a high proportion of 24 nt in-miRNAs in rice (Figures 3C and D) may indicate that this DNA methylation may be the main functional pathway for these in-miRNAs. When an in-miRNA mediates DNA methylation at its own locus, it actually directs methylation at the intronic region of its host gene. In our in-miRNA datasets, 65 (42%) have a potential to target their host genes. Osa-miR1852, miR2061.1, miR2061.3, and miR2061.4 even have two target sites in their host genes (Table S12). More interestingly, among the 54 lmiRNAs from Wu et al. (2010) [42], 31 (57%) were located in the intronic regions of protein-coding genes (Table S13). According to their experimental data, three in-miRNAs (e.g., osa-miR1863b, osa-miR1873.1, and osa-miR1876) could methylate their target genes, seven (e.g., osa-miR437-3p.2, osa-miR437-3p.3) could methylate their own loci, and osa-miR1873.1 could methylate both its own locus and target gene (Table S13).

Endogenous siRNAs could also direct DNA methylation at their own loci, which are often transposons or repetitive DNAs [48], [49]. To test whether DNA methylation triggered by endogenous siRNA is different from that triggered by in-miRNA, an expressed rice siRNA dataset, including 225 siRNAs (siRNA identification method from [37]), was extracted. Only 324 target genes were predicted for these siRNAs. Approximately 26% targets are related to transposon/retrotransposon genes to which ∼10% in-miRNA targets are related (Table S12; Table S14).

## Discussion

### Diversity of in-miRNAs and their Relationship to the Host Genes

In this study, we have identified 40 novel miRNAs derived from 20 intronic pre-miRNAs and 34 novel miRNAs from 18 annotated pre-miRNAs from the intronic regions of protein-coding genes of the rice genome using a comprehensive analysis of eight sets of small RNA sequencing data from the NCBI and gene annotation data from the MSU Rice Genome Annotation Project (Release 6.0; http://rice.plantbiologu.msu.edu) [28]. In addition, 79 known miRNAs should be attributed to in-miRNAs. All the 153 miRNAs derived from 89 pre-miRNAs residing in introns of protein-coding genes make up 29% of all rice miRNAs (536 in total).

Apart from the sizes, distribution patterns, and their organizations in the genomic structure (see Results), the in-miRNAs show abundant diversities in biogenesis, expression, function, and their relationship with host genes.

**Figure 6:**
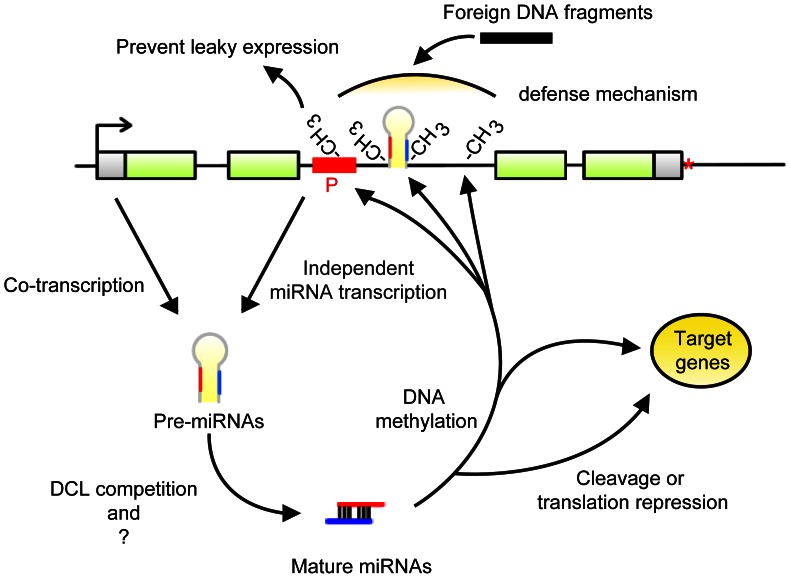
A model for biogenesis and in-miRNAs functions. The gray hairpin structure indicates pre-miRNA in which red and blue vertical lines represent miRNA and miRNA*, respectively. In the schematic diagram of the host gene structure, the red region with ‘P’ represents the independent promoter of in-miRNA. The red asterisk indicates the transcription terminal site. The CH_3_ located in the hairpin and adjacent intronic region indicate methylated regions.

In biogenesis, in-miRNAs present rich diversities due to the effect of host genes. First, from the transcription point of view, although previous reports have shown that in-miRNAs are transcribed along with host genes [11], [12], [13], some in-miRNAs may be regulated by their own upstream promoter. The independent transcription of in-miRNAs by their own promoters has been confirmed in humans and *C. elegans* [15], [16], . The upstream promoter-like sequences from rice pollen in-miRNAs can drive gene expression (data not shown). Therefore, rice in-miRNA-owned promoters provide another explanation for the complex expression relationships between in-miRNAs and host genes (Figure 6).

Second, from the perspective of mature processes of in-miRNAs, loci competition exists among DCL family members, such as between DCL1 and DCL3 [41], [42], in processing intronic MMP-miRNAs aside from alternative splicing. However, this case may occur in three-week-old rice seedlings specifically [42].

Furthermore, some long precursors can produce many miRNA-like sRNAs in a phased way (Table 4), as well as miRNAs through a precise cleavage (Table 2), indicating that some unknown mechanisms control this kind of cleavage.

In terms of functions, aside from the canonical pathway of miRNAs, in-miRNAs also play a role in DNA methylation (Figure 6) [42], [50]. In-miRNAs regulate more predicted targets but lesser transposon-related genes compared with siRNAs (Table S12; Table S14). In summary, in-miRNAs have their special functions in regulating gene expression and genome evolution.

### Function of in-miRNA-directed DNA Methylation

Methylation in the 5′ UTR mediated by in-miRNAs may result in the repression of the target gene transcription. For instance, an experiment validated miR1863 (an in-miRNA) and its target LOC_Os06g38480 [42]. As for the effect of DNA methylation by in-miRNAs on other transcribed regions, we can do the following analysis:

Zhang et al. (2006) reported that genes methylated in transcribed regions are highly expressed and constitutively active [51]. However, whether DNA methylation could directly affect gene expression is elusive [52], [53], [54]. Introns often need to face a large number of foreign DNA inserts [55]. A high proportion of intronic DNA methylation by in-miRNA can play an active role in preventing the insertion of foreign DNA fragments or leaky transcription of intragenic cryptic promoters (Figure 6) [56]. Thus, the expression levels of target genes are maintained by guarding the genomic sequence integrity and stabilizing transcription efficiency. Some studies show that intragenic methylation can also alter the chromatin structure and transcription elongation efficiency [50]. However, the effect on transcription elongation is limited because most mammalian genes contain methylated TE sequences within their introns [57]. Interestingly, considering the above viewpoints and the fact that in-miRNAs can target their own loci together, we may be able to interpret why host genes have a biased expression distribution. In-miRNA-directed self-DNA-methylation may maintain the expressions of their host genes and even protect their own integrity.

In conclusion, rice genomic introns recruit a large number of in-miRNAs that control thousands of coding gene expressions through post transcriptional and transcriptional gene silencing. These in-miRNAs have different expression patterns from their host genes and present a rich diversity in terms of structure, biogenesis, mechanism, and function. Our study provides a comprehensive analysis of rice in-miRNAs and enhances our understanding on their special functional role, especially in mediating DNA methylation, which was concluded to either temporarily repress gene expression or affect the stability of expression and gene structure (including host and target genes) in the evolution process.

## Supporting Information

Figure S1 **K-means Clustering of all intronic miRNAs.** All 153 intronic miRNAs were divided into 10 clusters according to their expression values. The sample labels: 1–5DAF, 6–10DAF, FLAGLEAF, 3WEEKSEEDLING, 4WEEKSEEDLING represent 1–5 day after fertilization (DAF), 6–10 DAF, flag leaves, 3 week seedlings, 4 week seedlings respectively. The expression values were normalized and then log2 transformed (see methods).
(TIF)

Figure S2 **Secondary Structure for novel intronic pre-miRNAs and two microRNA-like small RNAs.**
(PDF)

Figure S3 **Chip-seq data for host genes.** HY and CHD represent two sequencing samples. (A), (B) Dinstance indicates the distance between summit of the signal peak and the transcription start site of host gene. (C). LOC_Os03g52239 and its intronic MIR1188, the arrow indicates the summit of signal peaks. (D) LOC_Os08g41460 and its intronic osa-MIR2878.
(TIF)

Figure S4 **Twenty-six in-miRNAs with homologous fragments across eight plant species.** The block with light blue indicates in-miRNA have a homologous fragment in this plant species.
(TIF)

Figure S5 **GO enrichment analysis of target genes of rice intronic miRNAs.**
(TIF)

Table S1 **Descriptions of eight small RNA sequencing samples used in our study.**
(XLS)

Table S2 **Descriptions of microarray datasets used in our study.**
(XLS)

Table S3 The ratio of sequencing reads of miRNA/miRNA* duplex to that of its hairpin precursor for known miRNAs.
(DOC)

Table S4 The ratio of sequencing reads of miRNA/miRNA* duplex to that of its hairpin precursor for novel miRNAs and two microRNA-like small RNAs.
(DOC)

Table S5 **Primers of reverse transcriptase-PCR for pre-miRNA detection.**
(DOC)

Table S6 **Designed probes for liquid northern hybridization of mature miRNAs.**
(DOC)

Table S7 **Thirty-five hairpins considered as pre-miRNAs and two microRNA-like small RNAs.**
(XLS)

Table S8 **Mature miRNAs from known intronic pre-miRNAs.**
(XLS)

Table S9 **The normalized expression values of all in-miRNAs.**
(XLS)

Table S10 **Host genes containing in-miRNAs.**
(XLS)

Table S11 **Expression data and correlation analysis of in-miRNAs and their host genes.**
(XLS)

Table S12 **Predicted targets of in-miRNAs at DNA level.**
(XLS)

Table S13 **Long miRNAs studied by Wu et al (2010) located in introns.**
(XLS)

Table S14 **Predicted targets of rice siRNAs.**
(XLS)
