# Supplementary figures and images for: Genome-Wide Analysis Reveals Diversity of Rice Intronic miRNAs in Sequence Structure, Biogenesis and Function
Source: PLoS One. 2013 May 22;8(5):e63938. doi: 10.1371/journal.pone.0063938 (PMC3661559; doi:10.1371/journal.pone.0063938)

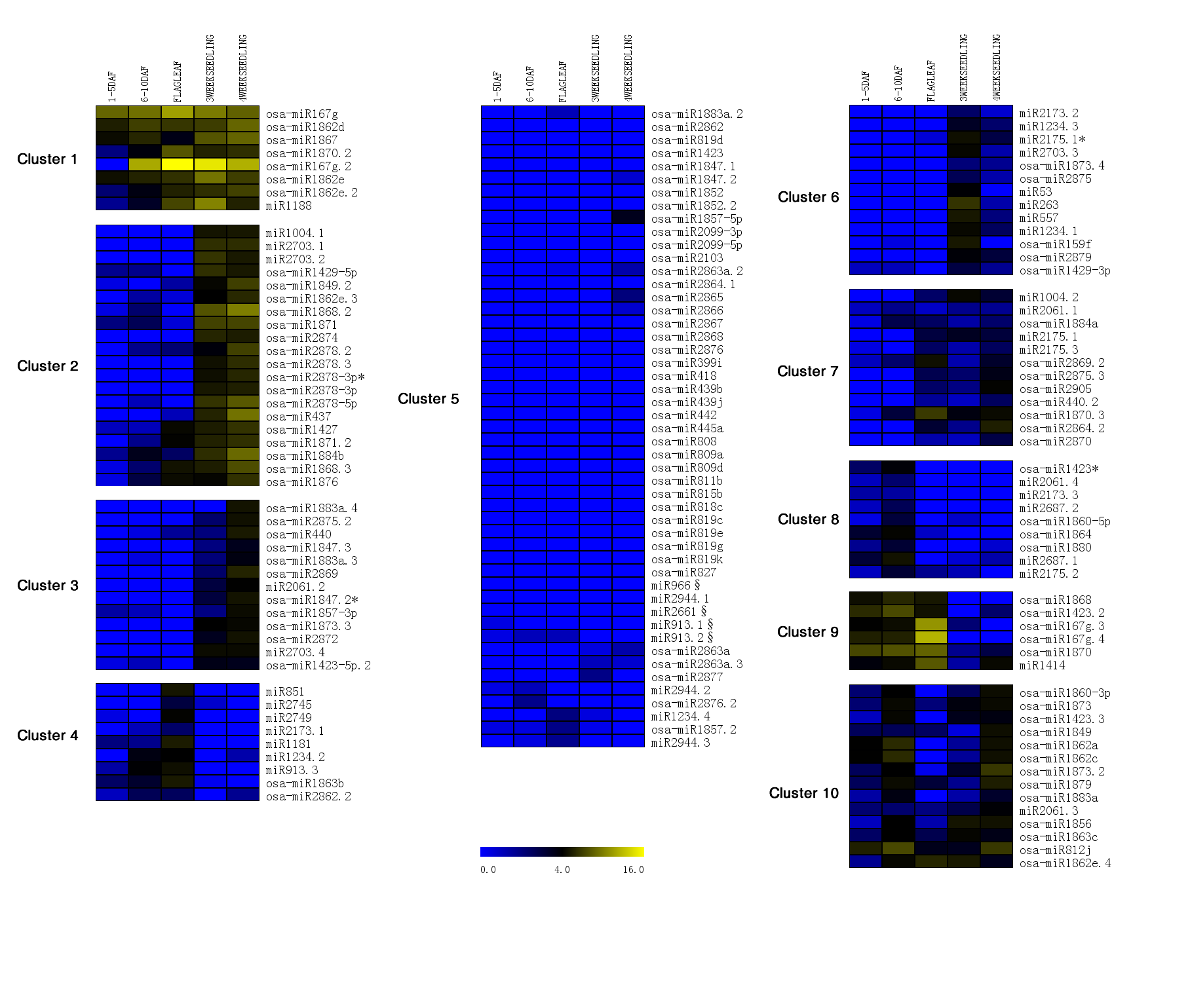

Supplement: Figure S1 — K-means Clustering of all intronic miRNAs. All 153 intronic miRNAs were divided into 10 clusters according to their expression values. The sample labels: 1–5DAF, 6–10DAF, FLAGLEAF, 3WEEKSEEDLING, 4WEEKSEEDLING represent 1–5 day after fertilization (DAF), 6–10 DAF, flag leaves, 3 week seedlings, 4 week seedlings respectively. The expression values were normalized and then log2 transformed (see methods). (TIF) [file pone.0063938.s001.tif]

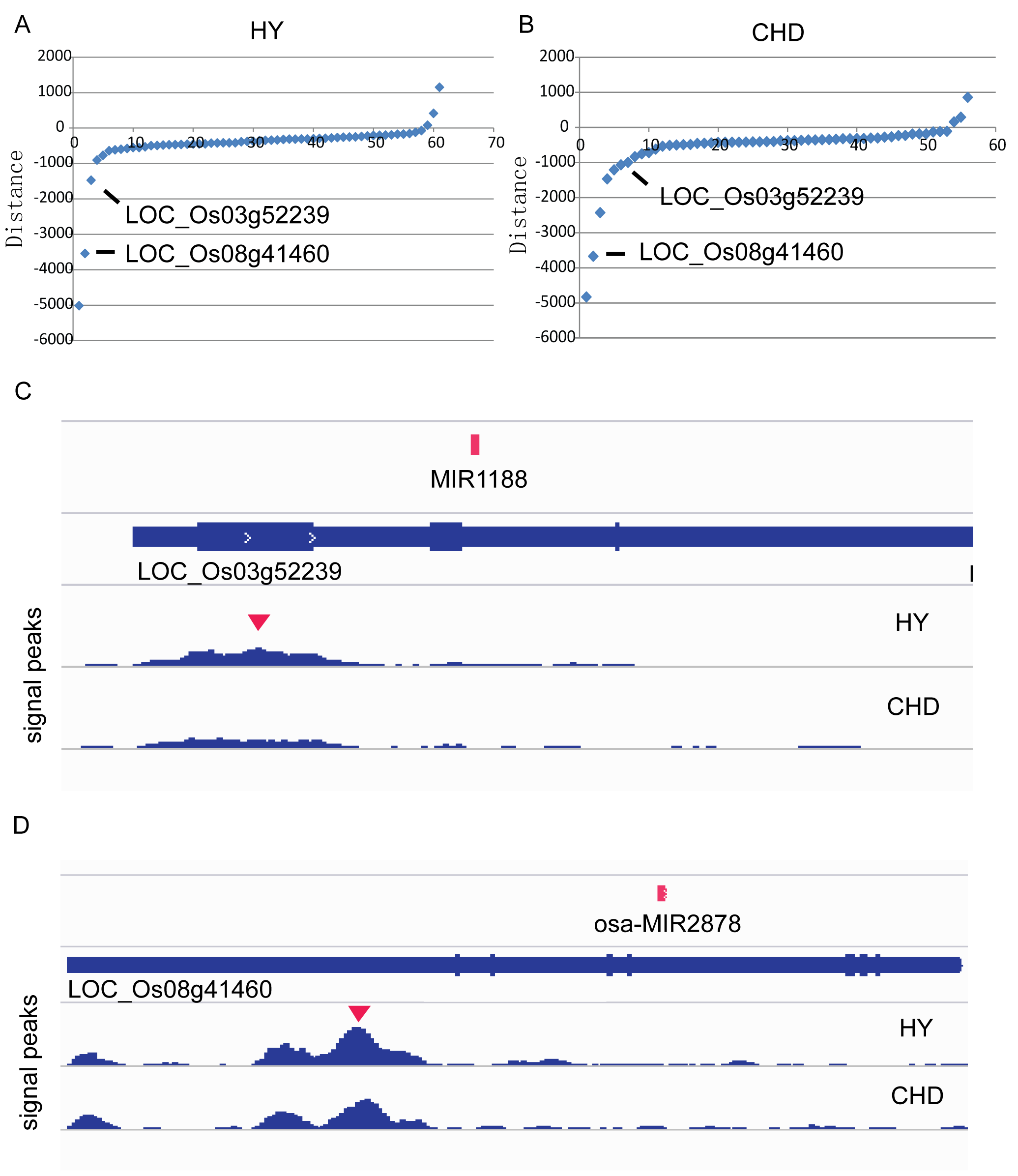

Supplement: Figure S3 — Chip-seq data for host genes. HY and CHD represent two sequencing samples. (A), (B) Dinstance indicates the distance between summit of the signal peak and the transcription start site of host gene. (C). LOC_Os03g52239 and its intronic MIR1188, the arrow indicates the summit of signal peaks. (D) LOC_Os08g41460 and its intronic osa-MIR2878. (TIF) [file pone.0063938.s003.tif]

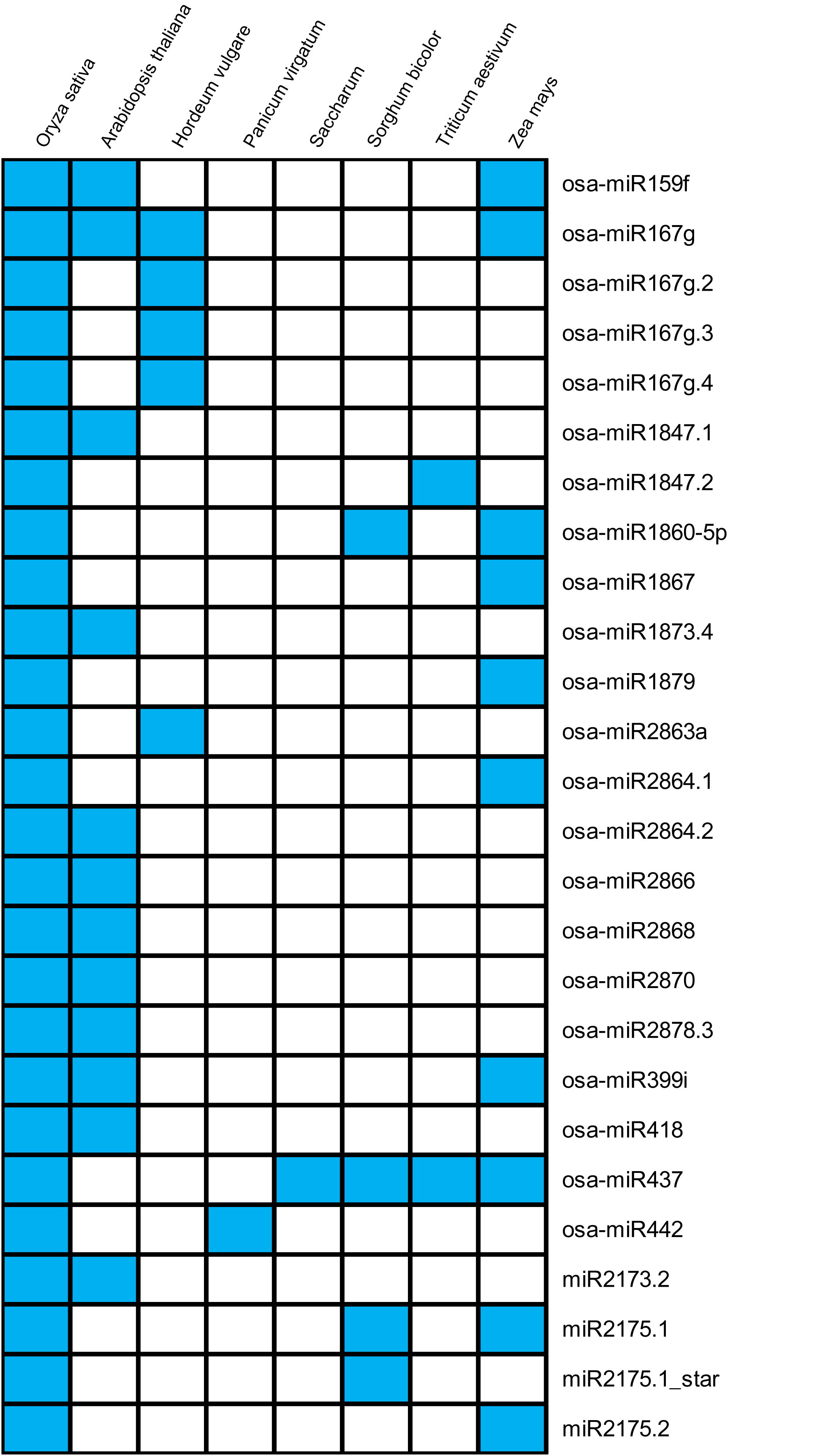

Supplement: Figure S4 — Twenty-six in-miRNAs with homologous fragments across eight plant species. The block with light blue indicates in-miRNA have a homologous fragment in this plant species. (TIF) [file pone.0063938.s004.tif]

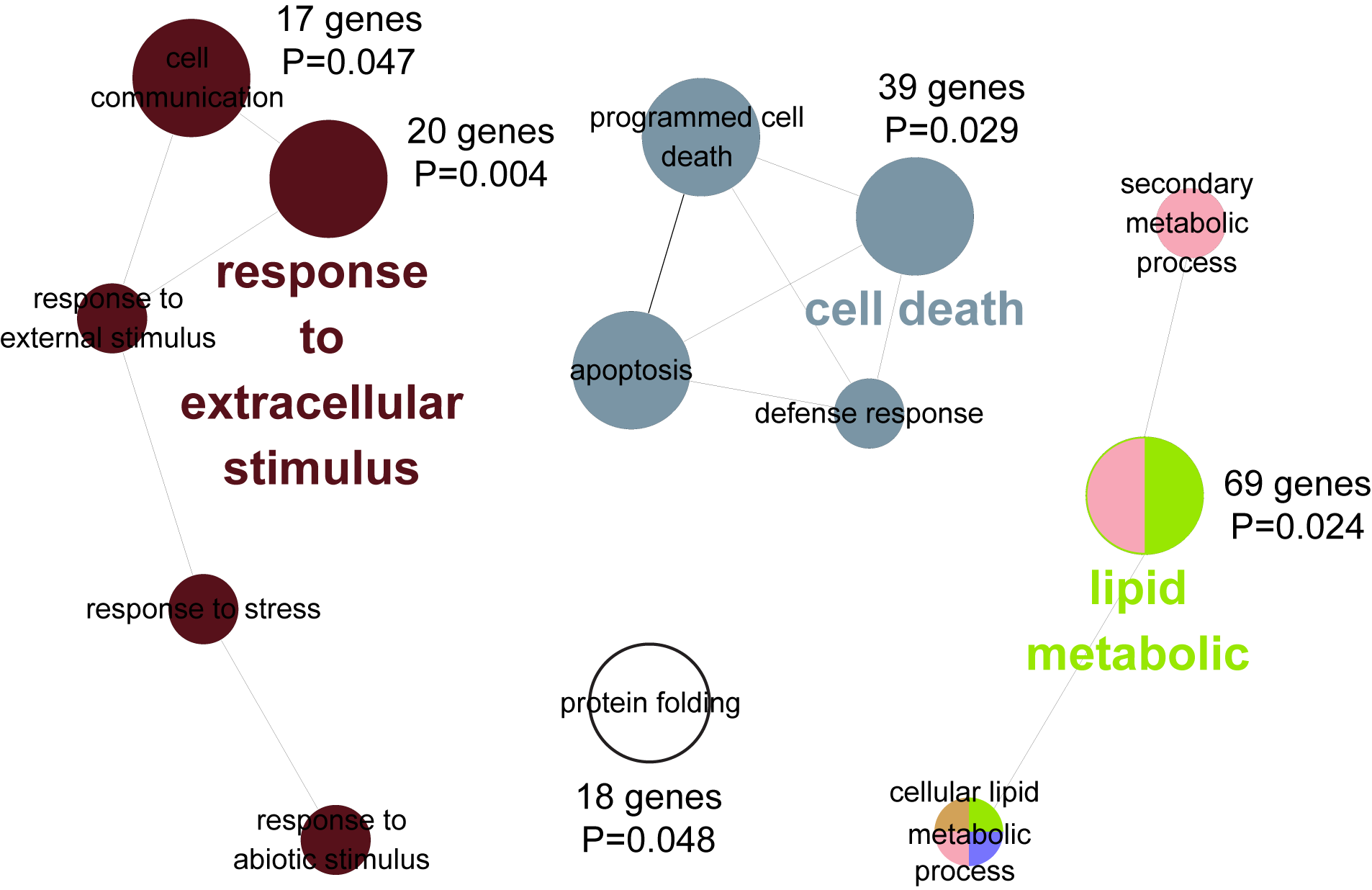

Supplement: Figure S5 — GO enrichment analysis of target genes of rice intronic miRNAs. (TIF) [file pone.0063938.s005.tif]
