# Supplementary material for: Genome-Wide Analysis Reveals Diversity of Rice Intronic miRNAs in Sequence Structure, Biogenesis and Function
Source: PLoS One. 2013 May 22;8(5):e63938. doi: 10.1371/journal.pone.0063938 (PMC3661559; doi:10.1371/journal.pone.0063938)
Supplement: Figure S2 — Secondary Structure for novel intronic pre-miRNAs and two microRNA-like small RNAs. (PDF) [file pone.0063938.s002.pdf]

pre-MIR53

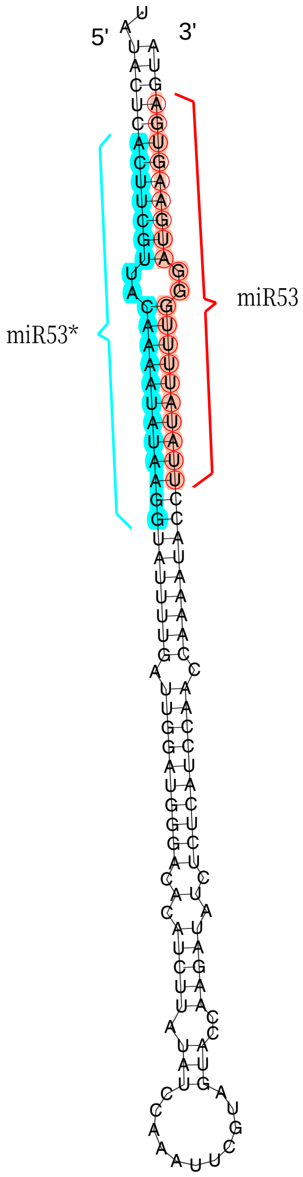

pre-MIR263

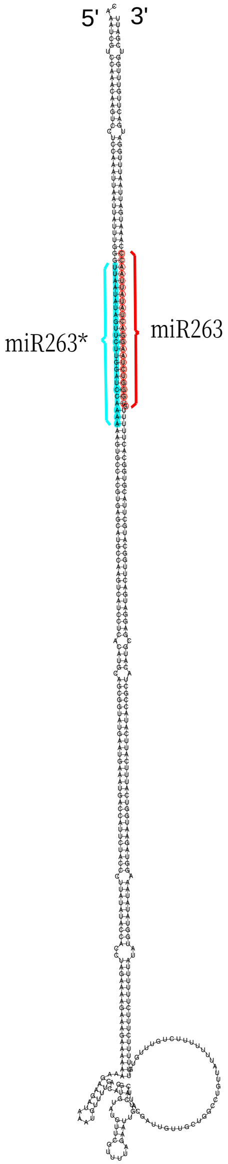

pre-MIR557

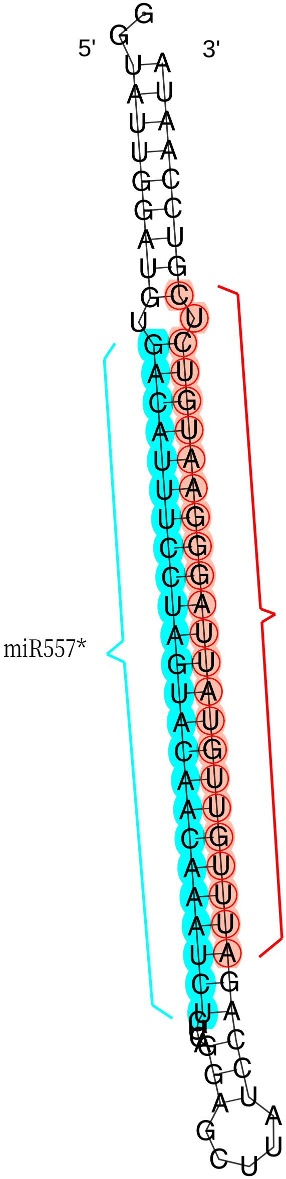

pre-MIR851

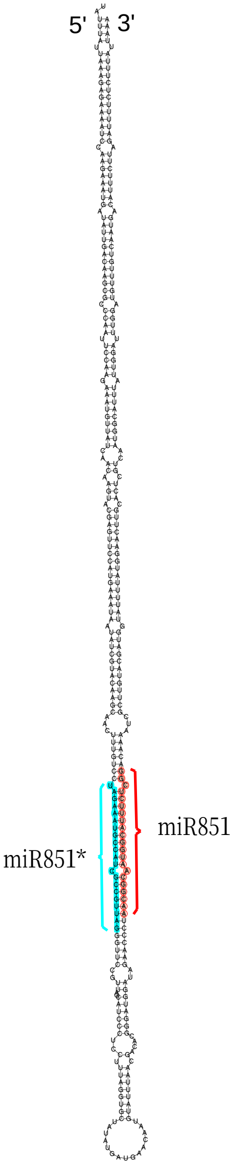

pre-MIR966

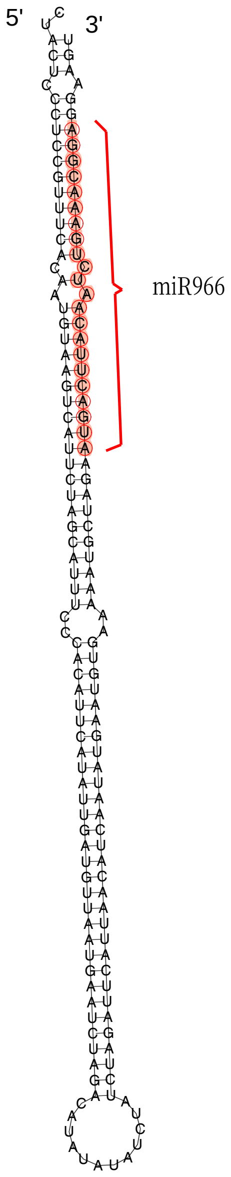

pre-MIR1188

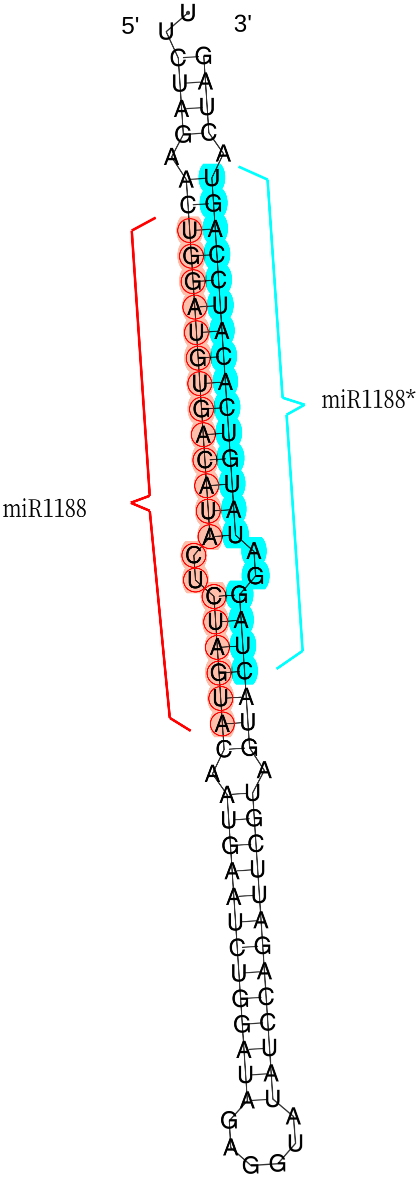

pre-MIR1414

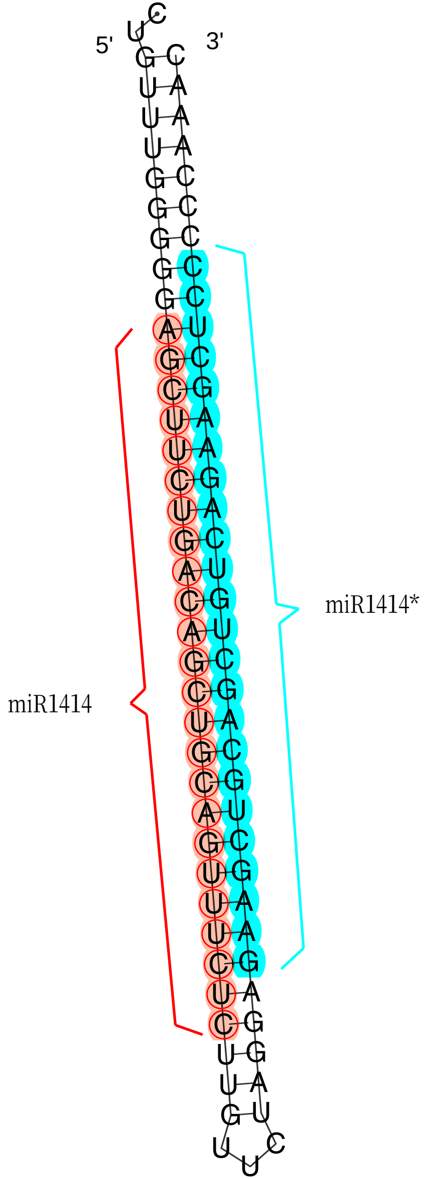

pre-MIR2173

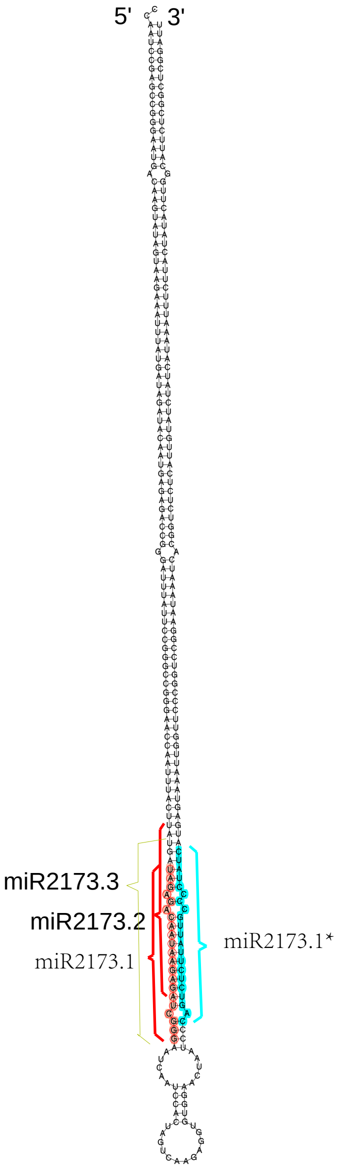

pre-MIR2175

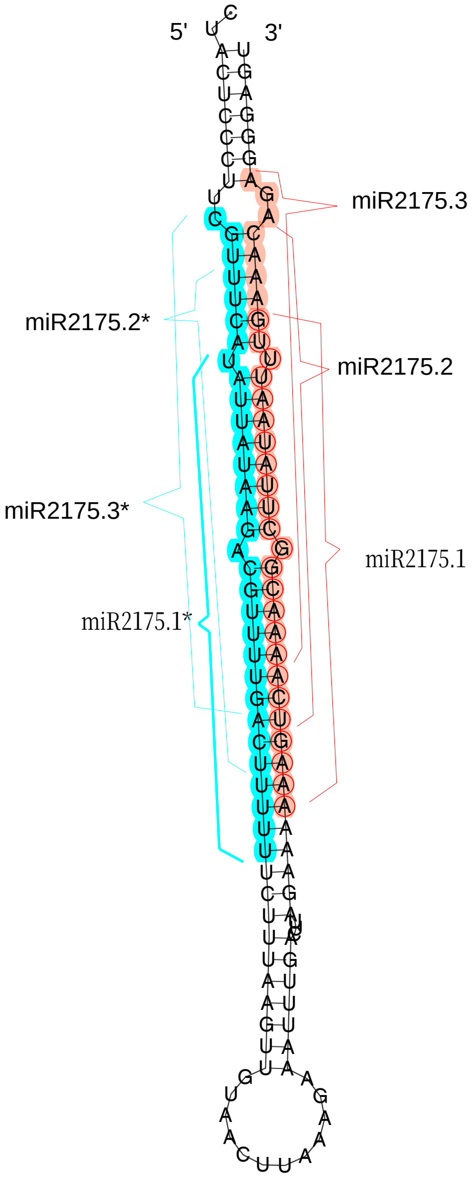
5'
3'
miR2175.3
miR2175.2*
miR2175.2
miR2175.3*
miR2175.1
miR2175.1*

pre-MIR2687

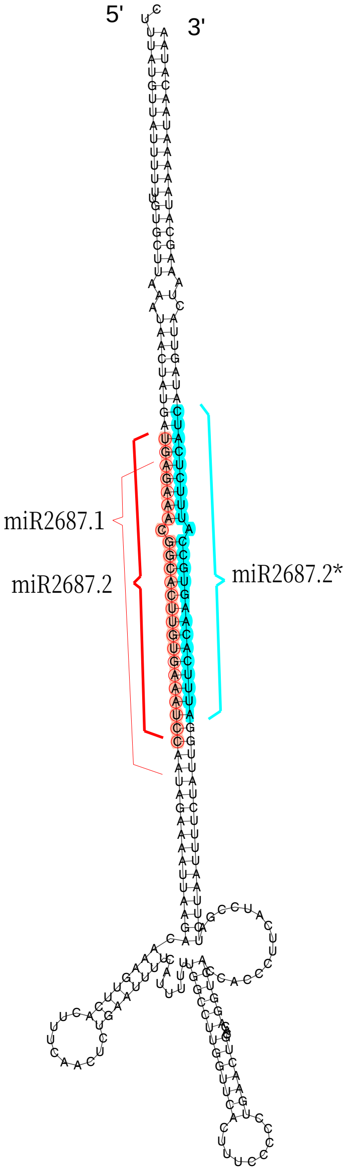

pre-MIR2745

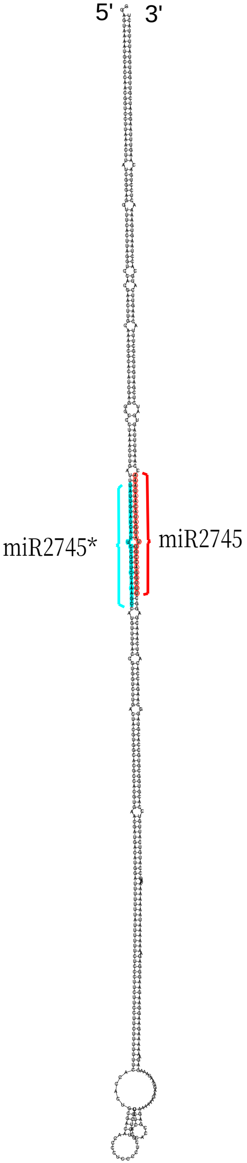

pre-MIR2749

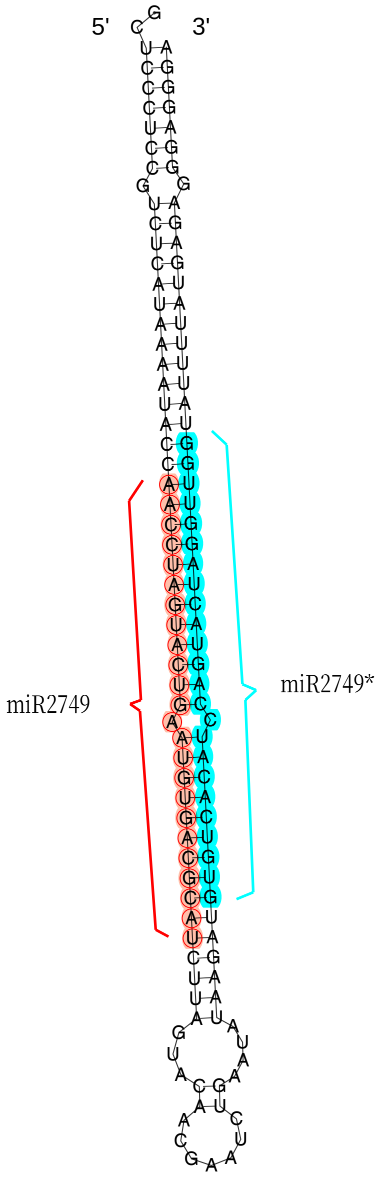

pre-MIR2944

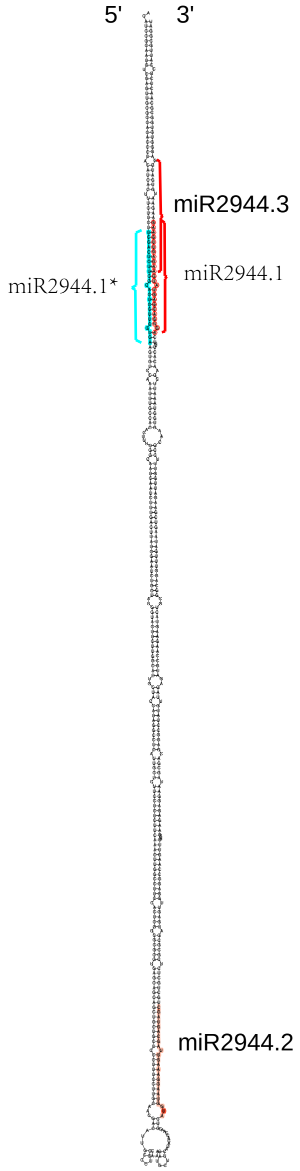

pre-MIR2661

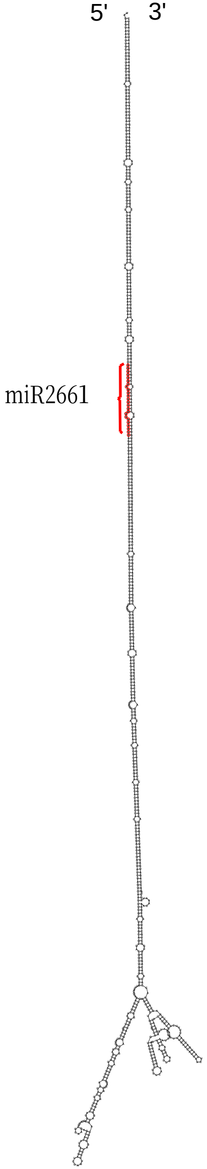

pre-MIR1181

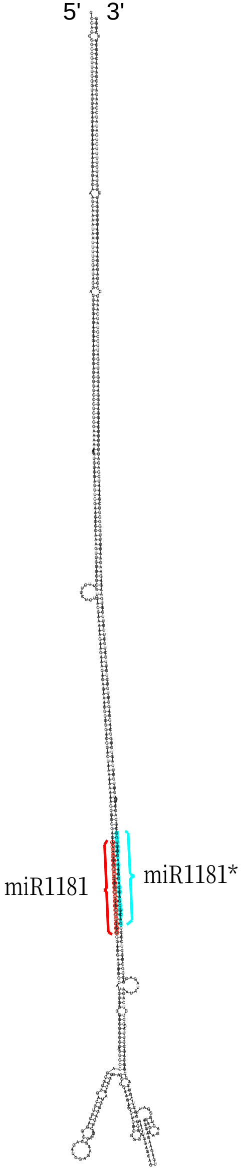

pre-MIR1004

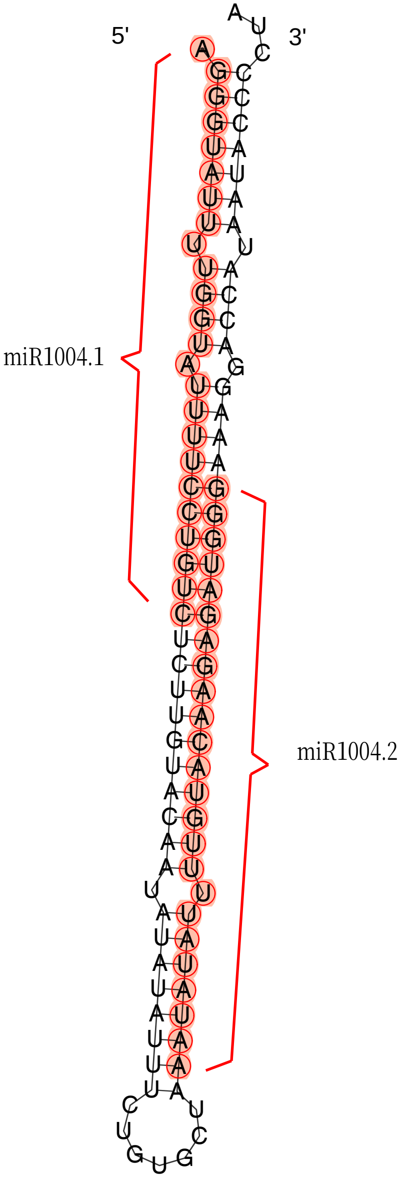

pre-MIR1234

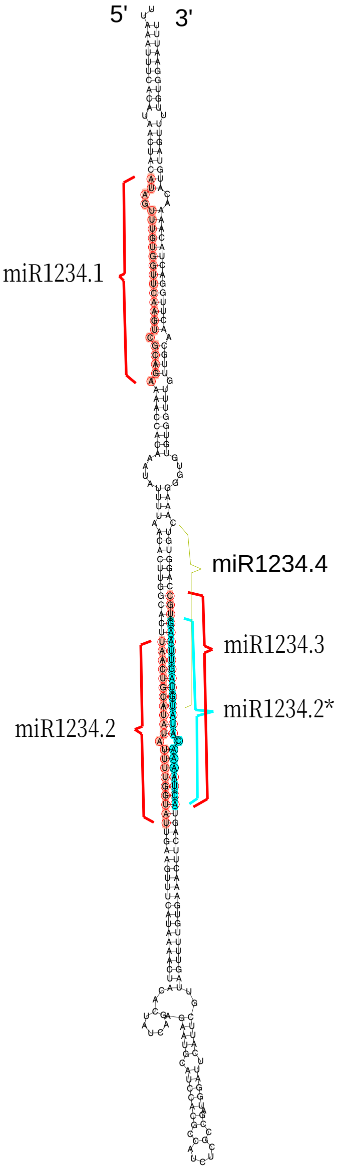

pre-MIR2061

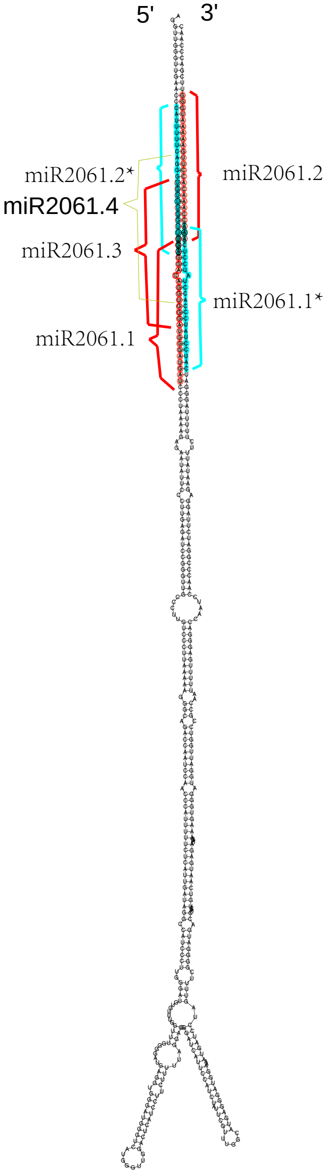
5'
3'
miR2061.2*
miR2061.4
miR2061.3
miR2061.1
miR2061.2
miR2061.1*

pre-MIR2703

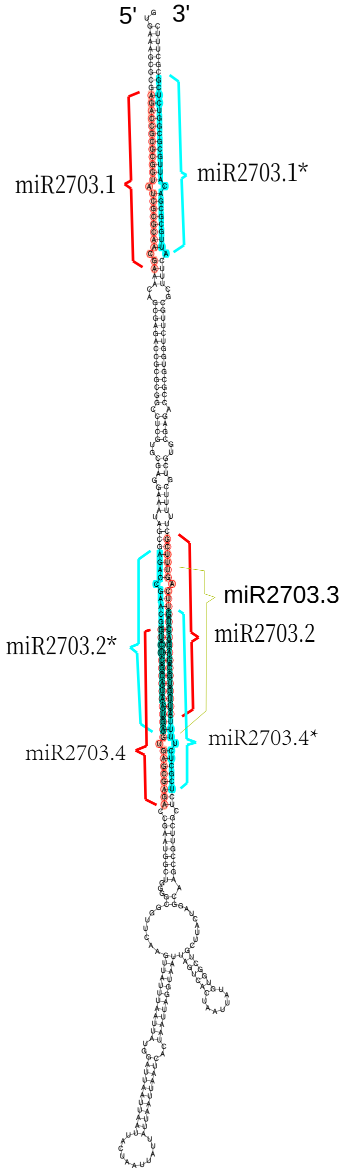

5'
3'
miR2703.1
miR2703.1*
miR2703.3
miR2703.2
miR2703.2*
miR2703.4
miR2703.4*

pre-MIR913

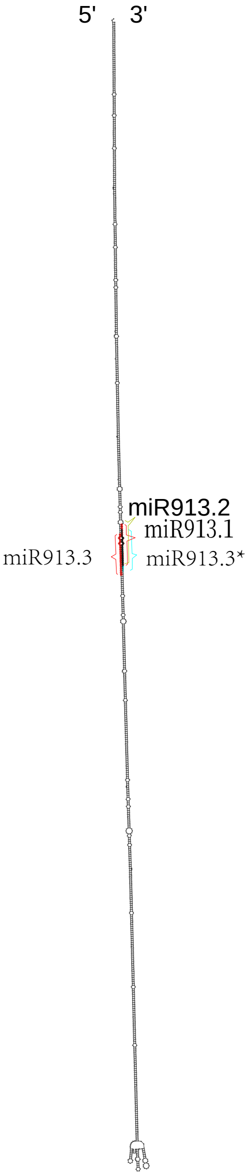

pre-MIR3863

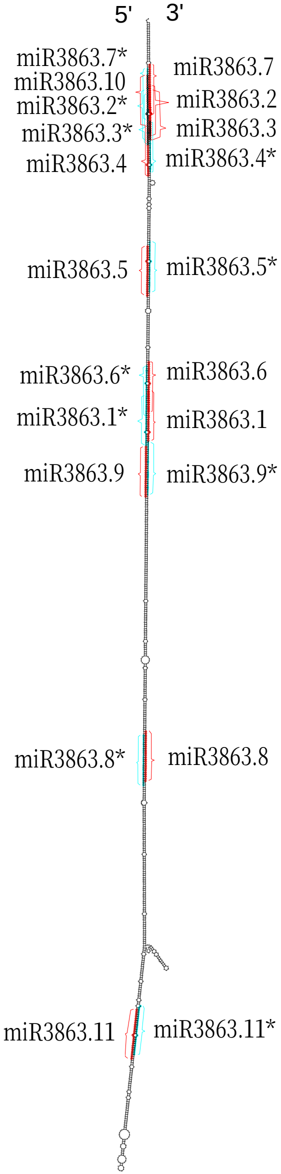
5'
3'
miR3863.7*
miR3863.7
miR3863.10
miR3863.2
miR3863.2*
miR3863.3
miR3863.3*
miR3863.4*
miR3863.4
miR3863.5
miR3863.5*
miR3863.6*
miR3863.6
miR3863.1*
miR3863.1
miR3863.9
miR3863.9*
miR3863.8*
miR3863.8
miR3863.11
miR3863.11*

pre-MIR2284

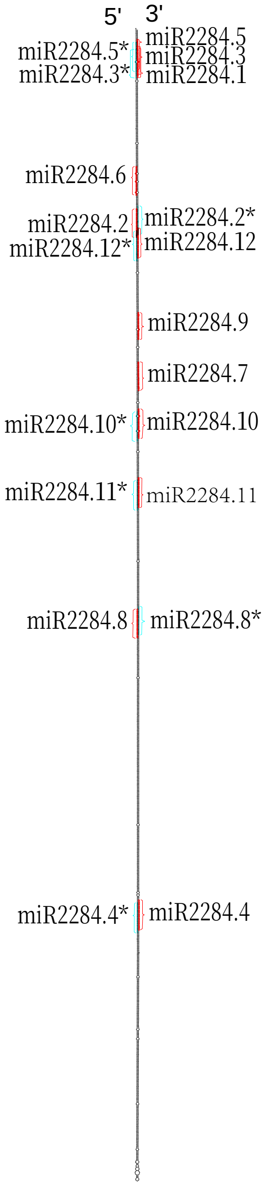

5'
3'
miR2284.5
miR2284.5*
miR2284.3
miR2284.3*
miR2284.1
miR2284.6
miR2284.2*
miR2284.2
miR2284.12
miR2284.12*
miR2284.9
miR2284.7
miR2284.10*
miR2284.10
miR2284.11*
miR2284.11
miR2284.8
miR2284.8*
miR2284.4*
miR2284.4
