# Supplementary material for: Genome-Wide Analysis Reveals Diversity of Rice Intronic miRNAs in Sequence Structure, Biogenesis and Function
Source: PLoS One. 2013 May 22;8(5):e63938. doi: 10.1371/journal.pone.0063938 (PMC3661559; doi:10.1371/journal.pone.0063938)
Supplement: Table S3 — The ratio of sequencing reads of miRNA/miRNA* duplex to that of its hairpin precursor for known miRNAs. (DOC) [file pone.0063938.s008.doc]

**Table S3. The ratio of sequencing reads of miRNA/miRNA* duplex to that of its hairpin precursor for known miRNAs.**

| **MiRNA** | **Mature miRNA reads** | **Hairpin reads** | **Reads ratio** |
| --- | --- | --- | --- |
| osa-mir531 | 2 | 2 | 100.00% |
| osa-mir418 | 1 | 1 | 100.00% |
| osa-mir394 | 32 | 32 | 100.00% |
| osa-mir1426 | 2 | 2 | 100.00% |
| osa-mir528 | 168177 | 168804 | 99.63% |
| osa-mir1878 | 1147 | 1167 | 98.29% |
| osa-mir1425 | 6913 | 7038 | 98.22% |
| osa-mir1318 | 64763 | 65986 | 98.15% |
| osa-mir535 | 39261 | 40176 | 97.72% |
| osa-mir1863 | 7733 | 7990 | 96.78% |
| osa-mir1433 | 186 | 196 | 94.90% |
| osa-mir1856 | 355 | 386 | 91.97% |
| osa-mir435 | 1383 | 1518 | 91.11% |
| osa-mir1877 | 270 | 299 | 90.30% |
| osa-mir408 | 11973 | 13348 | 89.70% |
| osa-mir1867 | 3240 | 3659 | 88.55% |
| osa-mir1876 | 508 | 600 | 84.67% |
| osa-mir1859 | 240 | 293 | 81.91% |
| osa-mir1427 | 661 | 827 | 79.93% |
| osa-mir1320 | 809 | 1029 | 78.62% |
| osa-mir390 | 782 | 996 | 78.51% |
| osa-mir393 | 147 | 189 | 77.78% |
| osa-mir2106 | 10 | 13 | 76.92% |
| osa-mir1880 | 63 | 94 | 67.02% |
| osa-mir440 | 146 | 226 | 64.60% |
| osa-mir1864 | 98 | 152 | 64.47% |
| osa-mir1881 | 181 | 302 | 59.93% |
| osa-mir1850.1, 2, 3^a^ | 3570+1979+0 | 5966 | 93.01% |
| osa-mir1847.1, 2^b^ | 0+108 | 183 | 59.02% |
| osa-mir1852 | 17 | 29 | 58.62% |
| osa-mir1875 | 170 | 301 | 56.48% |
| osa-mir1870 | 4177 | 7629 | 54.75% |
| osa-mir1871 | 1237 | 2347 | 52.71% |
| osa-mir1855 | 10 | 19 | 52.63% |
| osa-mir1439 | 1 | 2 | 50.00% |
| osa-mir442 | 1 | 2 | 50.00% |
| osa-mir1431 | 47 | 95 | 49.47% |
| osa-mir1423 | 1308 | 2688 | 48.66% |
| osa-mir1430 | 149 | 316 | 47.15% |
| osa-mir1872 | 34 | 76 | 44.74% |
| osa-mir1869 | 12 | 27 | 44.44% |

a: osa-miR1850.1, osa-miR1850.2 and osa-miR1850.3 have 3570 reads, 1979 reads and 0 reads, respectively.

b: osa-miR1847.2 have 108 reads, while osa-miR1847.1 have no reads.
