# Supplementary material for: Genome-Wide Analysis Reveals Diversity of Rice Intronic miRNAs in Sequence Structure, Biogenesis and Function
Source: PLoS One. 2013 May 22;8(5):e63938. doi: 10.1371/journal.pone.0063938 (PMC3661559; doi:10.1371/journal.pone.0063938)
Supplement: Table S4 — The ratio of sequencing reads of miRNA/miRNA* duplex to that of its hairpin precursor for novel miRNAs and two microRNA-like small RNAs. (DOC) [file pone.0063938.s009.doc]

**Table S4. The ratio of sequencing reads of miRNA/miRNA* duplex to that of its hairpin precursor for novel miRNAs and two microRNA-like small RNAs.**

| **ID** | **Ratio** |
| --- | --- |
| MIR53 | 73.9% |
| MIR263 | 89.3% |
| MIR557 | 85.9% |
| MIR851 | 75.2% |
| MIR966 | 81.6% |
| MIR1188 | 97.2% |
| MIR1414 | 94.5% |
| MIR2745 | 46.5% |
| MIR2749 | 49.5% |
| MIR2661 | 71.5% |
| MIR1181 | 42.3% |
| mir2944 | 66.3% |
| MIR2173 | 57% |
| mir1004 | 89.3% |
| miR2687 | 83.0% |
| MIR913 | 83.4% |
| MIR1234 | 55.1% |
| MIR2703 | 74.9% |
| MIR2061 | 65% |
| MIR2175 | 83.5% |
| MIR3863^a^ | 98.6% |
| MIR2284^a^ | 99.2% |

a: microRNA-like small RNAs.
