# Supplementary material for: Genome-Wide Analysis Reveals Diversity of Rice Intronic miRNAs in Sequence Structure, Biogenesis and Function
Source: PLoS One. 2013 May 22;8(5):e63938. doi: 10.1371/journal.pone.0063938 (PMC3661559; doi:10.1371/journal.pone.0063938)
Supplement: Table S5 — Primers of reverse transcriptase-PCR for pre-miRNA detection. (DOC) [file pone.0063938.s010.doc]

**Table S5. Primers of Reverse transcriptase-PCR for pre-miRNA detection.**

| **miRNA** | **Forward Primer** | **Reverse Primer** | **Product Length** |
| --- | --- | --- | --- |
| MIR1188 | TTCTAGAACTGGATGTGACA | CTACGAATCTGGATATACCT | 66 |
| MIR557 | GGTATTGGATGTGACATTTC | CGAGACATTCCCTAATACAA | 78 |
| MIR263 | CAAATCGTCCAAACAAGTCC | GCCAGCAACAATCGTAAGT | 229 |
| MIR2703 | GCTGGGGCGGTTCAAGTT | GTCAAAGCGAAAAAGCAGCA | 166 |
| MIR1004 | AGGGTATTTTGGTATTTTCC | GGTATTATGGTCCTTTCC | 91 |
| MIR2175 | CTACTCCCTTCGTTTCAT | CTCCCTCTGTTTCAAATTAT | 115 |
| MIR2061 | GGTTGCCCTTGTCCCTTA | ATCCCACTTTTCTGTCTCA | 229 |
| MIR913 | CATTACAGACCGATTCCGC | ACCTAACCCATCCCACCAG | 421 |
| MIR2661 | CCCACCGAGTTTGGCGTAT | TTGAGAGCCTCCCTTCCTG | 432 |
