# Supplementary material for: Genome-Wide Analysis Reveals Diversity of Rice Intronic miRNAs in Sequence Structure, Biogenesis and Function
Source: PLoS One. 2013 May 22;8(5):e63938. doi: 10.1371/journal.pone.0063938 (PMC3661559; doi:10.1371/journal.pone.0063938)
Supplement: Table S6 — Designed probes for liquid northern hybridization of mature miRNAs. (DOC) [file pone.0063938.s011.doc]

**Table S6. Designed Probes for Liquid Northern Hybridization of mature miRNAs.**

| **miRNA/Control** | **miRNA Sequence** | **Probe Sequence** | |
| --- | --- | --- | --- |
| miR1188 | UGGAUGUGACAUACUCUAGUA | (BIOTIN)-TACTAGAGTATGTCACATCCA |  |
| miR557 | AUUUGUUGUAUUAGGGAAUGUCUC | (BIOTIN)-GAGACATTCCCTAATACAACAAAT |  |
| miR263 | GUGGGUCUAAGGACUAUAUUAACC | (BIOTIN)-GGTTAATATAGTCCTTAGACCCAC |  |
| miR2703.1 | AGACCGCGCGGUAUCGCGCAACGA | (BIOTIN)-TCGTTGCGCGATACCGCGCGGTCT |  |
| miR1004.1 | AGGGUAUUUUGGUAUUUUCCUGUC | (BIOTIN)-GACAGGAAAATACCAAAATACCCT |  |
| miR2175.1* | UAUUAUAAGACGUUUUGACUUUUU | (BIOTIN)-AAAAAGTCAAAACGTCTTATAATA |  |
| miR2175.1 | AAAGUCAAAACGGCUUAUAAUUUG | (BIOTIN)-CAAATTATAAGCCGTTTTGACTTT |  |
| miR2061.3 | UGUUUGGUUUGAGGACAGGUGGGA | (BIOTIN)-TCCCACCTGTCCTCAAACCAAACA |  |
| miR913.1 | AUGGUACUGUAACCAGAAGCGG | (BIOTIN)-CCGCTTCTGGTTACAGTACCAT |  |
| miR2661 | AGGCUGUAGGAUUUGGGUUGAACC | (BIOTIN)-GGTTCAACCCAAATCCTACAGCCT |  |
| Control1^a^ | TGACAGAAGAGAGTGAGCAC (DNA) | (BIOTIN)-ACGTGCTCACTCTCTTCTGTCA |  |
| Control2 |  | (BIOTIN)-ACGTGCTCACTCTCTTCTGTCA |  |

a: Control1 is a probe-DNA hybrid duplex.
